# Combined computational, rational, and empirical design of boiling-resistant keratinase

**DOI:** 10.1128/aem.01860-25

**Published:** 2025-12-10

**Authors:** Yong Yang, Yuewen Luo, Yidi Ding, Yitong Yao, Jie Liu, Zinan Xu, Yu Li, Huai Li, Fei Gan, Xiao-Feng Tang, Bing Tang

**Affiliations:** 1 Hubei Key Laboratory of Cell Homeostasis, College of Life Sciences, Wuhan University 98436, Wuhan, China; 2 State Key Laboratory of Virology, College of Life Sciences, Wuhan University 98436, Wuhan, China; 3 Cooperative Innovation Center of Industrial Fermentation (Ministry of Education & Hubei Province), Wuhan, China; University of Milano-Bicocca, Milan, Italy

**Keywords:** subtilase, keratinase, thermostability, computational design, rational design, thermophile

## Abstract

Engineering of highly thermostable keratinase is of great theoretical interest in understanding protein stability mechanisms and practical significance for processing keratinous wastes such as feathers and wool. The thermostable subtilisin-like protease C2 is the major keratinase secreted by *Thermoactinomyces vulgaris* CDF but is rapidly inactivated at temperatures above 90°C. Here, we employed various methods to further stabilize protease C2. Four of the 35 selected single-point mutations designed by automated computational tools (PROSS, FireProt, ProteinMPNN, HyperMPNN, and ThermoMPNN) retained higher residual activity (~72%–84%) than protease C2 (~54%) after 1-h incubation at 85°C. The rational design of surface ion pairs and proline substitutions in β-turns generated two single-point variants with increased thermostability. Although three single-point aspartate substitutions appeared to be neutral, they could synergistically or cumulatively improve enzyme stability. The combination of these nine stabilizing mutations yielded the variant SM9 with a half-life of ~4 h at 100°C. The molecular dynamics simulations of protease C2 revealed several relatively flexible regions, including two Ca^2+^-binding sites (Ca1 and Ca2). Empirically modifying the Ca1 site and incorporating an additional two Ca^2+^-binding sites (Ca3 and Ca4) into the flexible regions yielded the variant CM1 with enhanced thermostability. By combining the mutations in SM9 and CM1, the variant CM16 was generated with a half-life of more than 9 h at 100°C. SM9 and CM16 are also highly resistant to high alkalinity, high salinity, urea, sodium dodecyl sulfate (SDS), organic solvents, and reductants, enabling them to efficiently degrade chicken feathers at temperatures near the boiling point.

**IMPORTANCE:**

The boiling-resistant enzymes are especially valuable not only for probing the molecular basis that allows proteins to function at the maximum temperature capable of supporting life but also offer the opportunity to greatly expand the enzymatic reaction conditions. Besides exploring naturally occurring boiling-resistant enzymes from hyperthermophiles, artificial engineering of enzymes with boiling resistance remains an important challenge. Our results demonstrate that the thermostability of the subtilisin-like protease C2 with keratinolytic activity can be largely improved by the combined use of automated computational design, structure-based rational design, and empirical engineering. The resulting variants are not only stable and functional at temperatures near or above 100°C but also show improved resistance to polyextreme conditions, providing new clues about the stabilization mechanism of subtilases. Moreover, by virtue of their hyperthermostability, the boiling-resistant variants could be repeatedly used for processing keratin substrates at high temperatures and find practical applications in feed, food, and leather industries.

## INTRODUCTION

Understanding the principles of protein stability is a long-standing yet challenging goal in biological science and is critical for designing enzymes with resistance to harsh industrial conditions, such as high temperature, high alkalinity or acidity, high salinity, and the presence of denaturants or organic solvents (1). The thermostable enzymes of (hyper)thermophiles have attracted great interest due to their contributions to the understanding of stabilization mechanisms of proteins and their potential for expanding the reaction conditions of biocatalysis (2, 3). In particular, hyperthermostable enzymes capable of functioning at temperatures near or above 100°C are especially valuable for probing molecular mechanisms of protein adaptation to the upper temperature limit for life (4). Meanwhile, the availability of an increasing number of hyperthermostable enzymes has opened new possibilities for industrial processes carried out at temperatures near the boiling point of water (2, 5).

Subtilisin-like serine proteases (subtilases) are widely distributed in bacteria, archaea, and eukaryotes and participate in important biological processes such as protein metabolism, nutrition, protein processing, and pathogen invasion (6, 7). They have been extensively studied not only to provide insights into the molecular basis for the structure-function relationship of enzymes but also due to their application potential in detergents, food and feed industry, leather industry, and medicine fields (8). Moreover, most microbial keratinases belong to subtilases, and thermostable keratinases are highly desired due to their high efficiency at high temperatures to degrade recalcitrant keratin with high degrees of cross-linking of disulfide bonds, hydrophobic interactions, and hydrogen bonds (9, 10). Several hyperthermophile-derived subtilases have been characterized to be stable at temperatures near or above 100°C and resistant to denaturants and detergents, such as aerolysin from *Pyrobaculum aerophilum* (11), pyrolysin from *Pyrococcus furiosus* (12, 13), STABLE protease from *Staphylothermus marinus* (14), Ap protease from *Aquifex pyrophilus* (15), pernisine from *Aeropyrum pernix* (16), Tk-subtilisin (17), and Tk-SP (18) from *Thermococcus kodakaraensis*, and GacK from *Geoglobus acetivorans* (19). In addition, islandisin from the thermophile *Fervidobacterium islandicum* is also highly stable at 100°C (20). In terms of application, pernisine (21), pyrolysin (22), Tk-subtilisin (23), Tk-SP (24), and GacK (19) can efficiently hydrolyze insoluble and recalcitrant proteins such as keratin and prion near or at 100°C (in some cases with the aid of denaturants), where the recalcitrant protein substrates will be destabilized and more susceptible to proteolysis (25). Moreover, the subtilase PfuS from *P. furiosus* (26) has been used for efficient protein fragmentation at high temperatures before the sequencing of proteins. Performing the enzymatic reactions at temperatures near or at the boiling point also has the advantages of accelerating reaction rates and decontaminating microbes and potential pathogens.

Besides exploring naturally occurring hyperthermostable enzymes, a few enzymes have been artificially engineered to be resistant to the boiling point of water. For instance, a moderately thermostable thermolysin-like protease TLP-ste from *Geobacillus stearothermophilus* was made hyperstable (resisting boiling) through replacing five residues with those of a more thermostable homolog of TLP-ste and introducing rationally designed mutations (a substitution of proline for serine and a disulfide bond) (27). By computationally guided design, an alcohol dehydrogenase from *Candida magnoliae* was engineered to approach boiling point stability (28). A mesophilic phytase from *Yersinia intermedia* has been engineered to be boiling-resistant either by evolution-guided design of N-glycosylation sites (29) or by rational design strategies including disulfide introduction, energy calculation, and B-factor analysis (30). Despite many efforts to improve enzyme stability, by far, there is no literature showing that a mesophilic or thermophilic subtilase has been engineered to be boiling-resistant.

Various approaches, including rational and computational design, phylogeny-based design, semi-rational design, and directed evolution, have been employed for engineering thermostable proteins (31). The recent successes of machine-learning prediction algorithms such as RoseTTAFold (32) and AlphaFold (33) allow the generation of highly accurate protein models, which not only facilitate structure-based rational protein engineering but also greatly promote the development of automated computational design tools to improve protein stability. For instance, PROSS (34) is a physical model-based strategy for designing multiple-point stabilizing mutations, and each of these mutations is predicted to be independently stabilizing. FireProt (35) is also a physical model-based method used for the design of either multiple- or single-point stabilizing mutations. ProteinMPNN (36) is a structure-based deep-learning approach to design new sequences predicted to fold into structures with improved soluble expression and thermostability, while retraining ProteinMPNN on proteins from hyperthermophiles generates the network HyperMPNN to design thermostable proteins (37). ThermoMPNN uses learned features extracted from ProteinMPNN to design single-point stabilizing mutations based on ΔΔG prediction (38). It would be interesting to investigate whether subtilases can be engineered to be boiling-resistant by using these newly emerging computational design tools.

*Thermoactinomyces vulgaris* CDF is a keratinolytic bacterium capable of degrading native chicken feathers at high temperatures (39, 40). We previously characterized a glutamyl endopeptidase (41), a spore-associated subtilase (protease CDF) (42), and two extracellular subtilases (protease C2 and Als) (39, 43) of strain CDF. The amino acid sequence of protease C2, which is the major keratinase of strain CDF, is identical to that of protease E79 from *Thermoactinomyces* sp. E79 (44), albeit with significant differences in the promoter regions of their genes. Protease C2 is a Ca^2+^-dependent, thermostable alkaline protease with an optimum temperature of 85°C at pH 10 (39) and shows tolerance to detergents, oxidants, and salinity (43, 45). In this report, we constructed stabilizing variants of protease C2 through automated computational design, structure-based rational design, and empirical engineering. The combination of the stabilizing mutations generated the variants with boiling resistance and enhanced polyextreme tolerance. The stabilizing mechanism of the enzyme was discussed, and the boiling-resistant variants were used to degrade native chicken feathers efficiently at high temperatures.

## RESULTS

### Automated computational design of variants with improved thermostability

**Fig 1:**
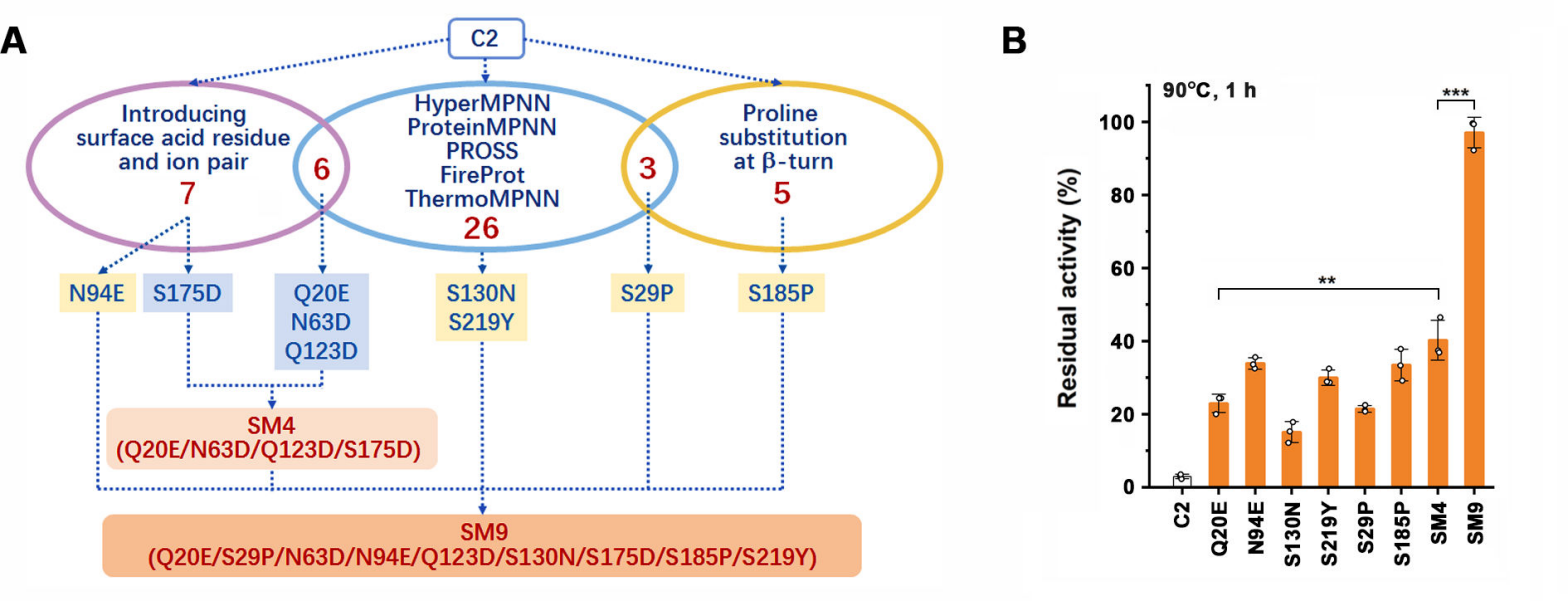
Thermostability of protease C2 and its variants designed by automated computational tools and structure-based rational approaches. (**A**) Schematic representation of the design of the variants. (**B**) Thermal resistance of the enzymes. The enzymes (1 µg/mL) were incubated at 90°C for 1 h in 50 mM Tris-HCl (pH 8) containing 10 mM CaCl_2_ and then subjected to azocaseinolytic activity assay at 60°C. The residual activity is expressed as a percentage of the original activity. The values are expressed as means ± SDs from three independent experiments (***P < 0.001; **P < 0.01; calculated by Student’s *t* test).

Our previous study shows that although protease C2 is thermostable, it rapidly loses activity at temperatures above 90°C (39). Here, we employed automated computational design tools, including PROSS, FireProt, ProteinMPNN, HyperMPNN, and ThermoMPNN, to further increase the thermostability of protease C2. Due to the variation in the algorithm, the five tools generated different mutant designs, where the number of predicted stabilizing mutations in different designs ranges from 7 to 119, and only one mutation (S182A) was predicted by all five tools (Table 1 ; Fig. S1). To improve the accuracy of selecting mutation candidates, we selected 26 mutations predicted by at least two tools (Table 1; Fig. S1). For comparison purposes, we also selected nine mutations predicted by only one tool; all of them are located on the enzyme surface, including three Asp substitutions, one Pro substitution, and five other types of substitutions (e.g., polar and aromatic) (Table 1; Fig. S1). In total, 35 single-point variants were constructed for experimental characterization (Fig. 1A and Table 1).

**TABLE 1:** Thermostabilities and activities of protease C2 and its variants designed by computational, rational, and empirical methods^*a*^

Automated computational design^*b*^					Enzyme^*c*^	Residual activity (%)	Relative activity (%)	Rational and empirical design	Enzyme^*c*^	Residual activity (%)	Relative activity (%)
H	P	T	R	F							
					C2	54.41 ± 4.83	100.00 ± 12.49	Proline substitution at b-turn	T42P	38.59 ± 3.23 (*)	85.36 ± 4.57 (n.s.)
√	√				**Q20E**	71.80 ± 7.35 (*)	109.56 ± 3.64 (n.s.)		N105P	52.29 ± 4.83 (n.s.)	72.04 ± 1.79 (*)
	√	√	√	√	S27G	50.50 ± 1.67 (n.s.)	87.23 ± 9.21 (n.s.)		A172P	54.21 ± 4.23 (n.s.)	64.78 ± 1.71 (*)
√	√				**S29P**	83.83 ± 2.24 (**)	86.40 ± 1.96 (n.s.)		**S185P**	87.19 ± 4.02 (**)	92.98 ± 2.72 (n.s.)
	√	√			K50R	3.36 ± 1.12 (***)	108.13 ± 1.40 (n.s.)		Y191P	65.36 ± 4.59 (n.s.)	94.30 ± 4.60 (n.s.)
	√	√			D56N	54.41 ± 2.15 (n.s.)	90.70 ± 5.31 (n.s.)	Introducing surface acid residue and ion pair	**N94E**	92.32 ± 6.85 (**)	93.89 ± 4.23 (n.s.)
√	√				D61N	45.33 ± 4.81 (n.s.)	117.49 ± 2.82 (n.s.)		**N94D**	80.23 ± 6.94 (*)	84.16 ± 8.42 (n.s.)
			√		N63D	62.54 ± 3.98 (n.s.)	110.68 ± 3.24 (n.s.)		S163E	66.70 ± 7.83 (n.s.)	126.11 ± 14.84 (n.s.)
√			√	√	C74V	56.77 ± 4.31(n.s.)	64.26 ± 4.25 (*)		S175D	63.14 ± 5.98 (n.s.)	116.11 ± 21.10 (n.s.)
√	√	√	√		N104D	66.37 ± 5.72 (n.s.)	97.00 ± 2.76 (n.s.)		S175E	63.41 ± 5.55 (n.s.)	105.23 ± 3.93 (n.s.)
			√		N105D	52.29 ± 4.83 (n.s.)	64.15 ± 6.31 (*)		Y191D	62.40 ± 4.75 (n.s.)	108.66 ± 1.51 (n.s.)
√	√	√			S106N	49.24 ± 2.15 (n.s.)	76.54 ± 12.99 (n.s.)		N207D	62.73 ± 12.48 (n.s.)	82.77 ± 16.78 (n.s.)
√	√	√			M111L	56.77 ± 4.31 (n.s.)	111.36 ± 4.94 (n.s.)	Modifying and incorporating Ca^2+^-binding sites	D59N	40.88 ± 3.83 (*)	80.16 ± 3.41 (n.s.)
			√		A112S	62.03 ± 5.25 (n.s.)	102.84 ± 4.55 (n.s.)		V81D	6.91 ± 2.03 (***)	101.52 ± 14.69 (n.s.)
√		√	√		V114I	58.79 ± 2.05 (n.s.)	84.90 ± 13.12 (n.s.)		V81N	28.95 ± 2.24 (**)	58.79 ± 15.54 (*)
		√	√		A119I	57.21 ± 2.16 (n.s.)	71.89 ± 2.92 (*)		V81E	41.94 ± 3.47 (*)	99.06 ± 0.93 (n.s.)
			√		Q123D	56.35 ± 6.22 (n.s.)	110.22 ± 1.67 (n.s.)		V81Q	24.64 ± 4.80 (**)	75.72 ± 13.45 (n.s.)
√	√		√	√	**S130N**	78.55 ± 1.88 (**)	101.30 ± 2.90 (n.s.)		G85D	21.83 ± 0.30 (***)	96.36 ± 2.46 (n.s.)
√	√				G135S	46.55 ± 9.27 (n.s.)	79.37 ± 4.60 (n.s.)		G85N	35.37 ± 2.94 (**)	73.77 ± 3.00 (*)
√	√	√			S137T	63.57 ± 5.07 (n.s.)	76.44 ± 16.12 (n.s.)		G85E	2.21 ± 0.20 (***)	88.75 ± 5.07 (n.s.)
			√		A141T	55.65 ± 3.42 (n.s.)	88.09 ± 3.90 (n.s.)		G85Q	2.83 ± 0.66 (***)	61.22 ± 15.77 (n.s.)
			√	√	Q148Y	54.48 ± 1.25 (n.s.)	82.48 ± 1.55 (n.s.)		T86D	23.09 ± 0.72 (***)	72.55 ± 5.72 (*)
√	√		√	√	S152K	51.31 ± 1.50 (n.s.)	71.49 ± 7.58 (n.s.)		T86N	11.13 ± 0.56 (***)	68.35 ± 5.55 (*)
	√		√		S163D	59.29 ± 3.01 (n.s.)	114.45 ± 8.26 (n.s.)		**T86E**	82.97 ± 0.32 (**)	89.63 ± 4.86 (n.s.)
	√		√		S164N	50.91 ± 3.32 (n.s.)	67.99 ± 10.99 (n.s.)		T86Q	60.52 ± 6.47 (n.s.)	62.78 ± 5.88 (*)
√	√		√		S175P	56.14 ± 1.59 (n.s.)	71.42 ± 1.75 (*)		**Q48D**	68.51 ± 3.06 (*)	98.53 ± 4.13 (n.s.)
			√		Q176N	55.70 ± 3.24 (n.s.)	77.55 ± 1.77 (n.s.)		**Ca3M**	96.97 ± 5.17 (**)	90.03 ± 2.27 (n.s.)
		√	√		A177V	58.02 ± 3.02 (n.s.)	63.85 ± 5.12 (*)		**Ca4M**	83.16 ± 2.92 (**)	75.56 ± 3.26 (n.s.)
√	√	√	√	√	S182A	48.22 ± 2.00 (n.s.)	103.90 ± 4.02 (n.s.)		Ca5M	0.52 ± 1.15 (***)	59.52 ± 1.61 (*)
√			√	√	S190A	9.16 ± 1.05 (***)	85.20 ± 3.10 (n.s.)				
	√		√		Y191S	61.84 ± 4.16 (n.s.)	107.68 ± 4.09 (n.s.)				
√					S197P	4.05 ± 1.36 (***)	71.31 ± 1.67 (*)				
√			√		S206V	36.44 ± 2.04 (**)	91.89 ± 5.82 (n.s.)				
	√				Y212W	64.13 ± 3.40 (n.s.)	69.56 ± 9.23 (n.s.)				
			√		**S219Y**	77.53 ± 2.18 (**)	89.61 ± 2.71 (n.s.)				
	√		√		L220M	57.65 ± 5.73 (n.s.)	65.74 ± 9.36 (*)				

^*a*^ The enzymes (1 μg/mL) were incubated at 85°C for 1 h in 50 mM Tris-HCl (pH 8) containing 10 mM CaCl_2_ and then subjected to azocaseinolytic activity assay at 60°C. The residual activity is expressed as a percentage of the original activity. The original activities of the enzymes were used to calculate the relative activity with the original activity of protease C2 defined as 100%. The values are expressed as means ± SDs from three independent experiments (***P <0.001; **P < 0.01; *P < 0.05; n.s., no significance; calculated by Student’s *t* test).

^*b*^ The mutations predicted by HyperMPNN (H), ProteinMPNN (P), ThermoMPNN (T), PROSS (R), or FireProt (F) are indicated by “√”.

^*c*^ The variants with increased thermostability are bolded.

The proforms of protease C2 and its variants were expressed in *Escherichia coli* and heat treated for autocatalytic processing and degradation of the N-terminal propeptide, and the resulting mature forms were purified (Fig. S2). The thermostabilities of the enzymes were investigated by measuring their residual activities at 60°C after heat treatment at 85°C for 1 h. Compared with protease C2, which retained ~54% residual activity after the heat treatment, 4 (Q20E, S29P, S130N, and S219Y) of the 35 single-point variants showed significantly higher residual activity (~72%–84%) while the other 31 variants exhibited similar or lower residual activity (Table 1). We noticed that none of the four stabilizing mutations was predicted by ThermoMPNN (Table 1), which is used for the design of thermostable proteins with single-point mutation (38). Among the 35 experimentally tested mutations, 17, 20, 24, and 7 were predicted by HyperMPNN, ProteinMPNN, PROSS, and FireProt, respectively; for the four confirmed stabilizing mutations, S130N was predicted by all four computational tools, and Q20E and S29P were predicted by both HyperMPNN and ProteinMPNN, while S219Y was predicted only by PROSS (Table 1).

It is possible that individual mutations contribute little to stability, and making multiple-point mutations may markedly improve protein stability (46). To test this possibility, the low-risk multiple-point design of FireProt, which contains seven mutations (L6F/S27G/C74V/A113W/Q148Y/S164G/S182A; named FP7), was constructed and expressed in *E. coli*. Although the proform of FP7 in the soluble cellular fraction could convert to mature form by autoprocessing its N-terminal propeptide, after incubation at 85°C for 15 min both the pro- and mature forms were precipitated while the processed N-terminal propeptide remained soluble (Fig. S3). By contrast, after heat treatment, the proform of protease C2 converted to soluble mature form, and the mature form exhibited proteolytic activity to degrade the processed N-terminal propeptide and the proteins of *E. coli* cells (Fig. S3). These results suggest that the variant FP7 is less thermostable than protease C2.

### Structure-based rational design of surface ion pairs and Pro substitution to enhance enzyme thermostability

Hyperthermostable subtilases usually have an overall excess of acidic residues (Asp and Glu) involved in forming ion pairs, Ca^2+^-binding sites, and hydration shell at elevated salinity (14–18, 20, 47). Solvent-accessible ion pairs, including salt bridges and long-range ion pairs, represent important stabilizing forces of hyperthermostable proteins (4, 48). According to the structure model of protease C2 (Fig. S4), ion pairs of the enzyme were analyzed by Visual Molecular Dynamics (VMD) software (49), with a distance limit of 4 Å and 8 Å defined as a salt bridge (50) and a long-range ion pair (48), respectively. In total, 13 ion pairs (8 salt bridges and 5 long-range ion pairs) were identified, and 12 of them form 3 ionic networks (Table S1). The solvent accessibility of the residues (Table S2) and ion pairs (Table S3) of protease C2 was analyzed by calculating their relative solvent accessible surface area (RASA) values using GetArea (51), revealing seven exposed ion pairs (three salt bridges and four long-range ion pairs) (Table S3) with RASA value higher than 20% (52). We selected the residues Lys15 (forming the surface salt bridge K15-D187 and the surface long-range ion pair K15-D184) and Arg26 (forming the surface long-range ion pair D23-R26) (Table S3) for mutational analysis, and the resulting variants K15A and R26A showed significantly lower residual activities than protease C2 after 1-h incubation at 85°C (Fig. S5), suggesting that the selected surface ion pairs may contribute to thermostability of the enzyme. For the six selected Asp/Glu substitution variants designed by automated computational tools, additional ion pairs are formed in N104D, N105D, and Q123D but not in Q20E, N63D, and S163D (Table S1). Among the six variants, Q20E showed a significantly higher thermostability than protease C2 (Table 1).

We next investigated whether the thermostability of protease C2 could be improved by introducing additional surface ion pairs and acidic residues. Seven (Lys15, Arg26, Arg101, Arg248, Lys257, Arg269, and Lys274) of the 10 positively charged residues of protease C2 participate in the ion pair formation (Table S1), whereas the other three (Arg9, Lys50, and Arg242) do not. The solvent-accessible surface residues (Table S2) surrounding these positively charged residues were subjected to *in silico* analysis for their potential to form additional ion pairs if mutated to negatively charged residues. Finally, we selected the surface residues Asn94 and Ser175 for Asp/Glu substitution (N94D, N94E, S175D, and S175E) to form new ion pairs with Arg26, Lys50, and Arg248 (Table S3). The surface residues Ser163, Tyr191, and Asn207 (Table S2), which are far from the 10 positively charged residues in protease C2, were also selected for Asp/Glu substitution (S163E, Y191D, and N207D) to introduce additional surface acidic residues. The mutation Y191D does not establish an ion pair between Asp191 and any of the positively charged residues but leads to the formation of additional ion pairs at distant sites (D5-R9 and R9-D46) (Table S1). The experimental data showed that only N94D and N94E among the seven variants retained a significantly higher residual activity than protease C2 after heat treatment at 85°C for 1 h (Table 1). These results imply that the positions of acidic residues and ion pairs correlate with their roles in stabilizing the enzyme.

Although several Asp/Glu substitutions individually appeared to be neutral (Table 1), they may possibly stabilize the enzyme in a cumulative or synergistic manner. To test this possibility, three single-point substitutions (N63D, Q123D, and S175D), which did not lead to a significant change in thermostability at 85°C (Table 1) and are far away from the active site (Fig. S4), were selected and combined with Q20E to generate the variant SM4 (Fig. 1A). Compared with Q20E, SM4 displayed a significantly higher residual activity after heat treatment at 90°C for 1 h (Fig. 1B), implying a cumulative or synergistic role of the three Asp substitutions in stabilizing the enzyme.

Proline is also regarded as an important contributor to protein thermostability (4), and Pro substitution at the *i* + 1 position of Type I β-turn can increase protein stability by decreasing the entropy of unfolding (53). According to the structure model of protease C2, there are 12 Type I β-turns, of which two naturally contain a proline at the *i* + 1 position, and one is a Ca^2+^-binding site (Table S4). In the three Pro substitution variants (S29P, S175P, and S197P) designed by computational tools, the mutation sites are all located at the *i* + 1 positions of three Type I β-turns, respectively (Table S4), while S29P was confirmed to be more thermostable than protease C2 (Table 1). We then constructed additional variants (T42P, N105P, A172P, S185P, Y191P, and Q266P) with Pro substitutions at the *i* + 1 positions of the remaining 6 Type I β-turns (Table S4), but only S185P showed an increased thermostability (Table 1). Among the nine Pro substitution variants constructed here, T42P and S197P showed a decreased thermostability (Table 1), while Q266P was even less stable than the two variants, as evidenced by the observation that Q266P suffered from severe autodegradation (Fig. S2).

The stabilizing mutations (S29P, N94E, S130N, S185P, and S219Y) were introduced into SM4, yielding the variant SM9 (Fig. 1A). Compared with SM4 and the single-point variants with a stabilizing mutation, SM9 showed significantly higher thermostability and retained 97.0% residual activity after incubation at 90°C for 1 h (Fig. 1B), reflecting the cumulative effect of the stabilizing mutations. Additionally, all of the variants with improved thermostability exhibited an activity similar to protease C2 at 60°C (Table 1).

### Sequence alignment and MD simulations identify the regions for enhancement of thermostability

**Fig 2:**
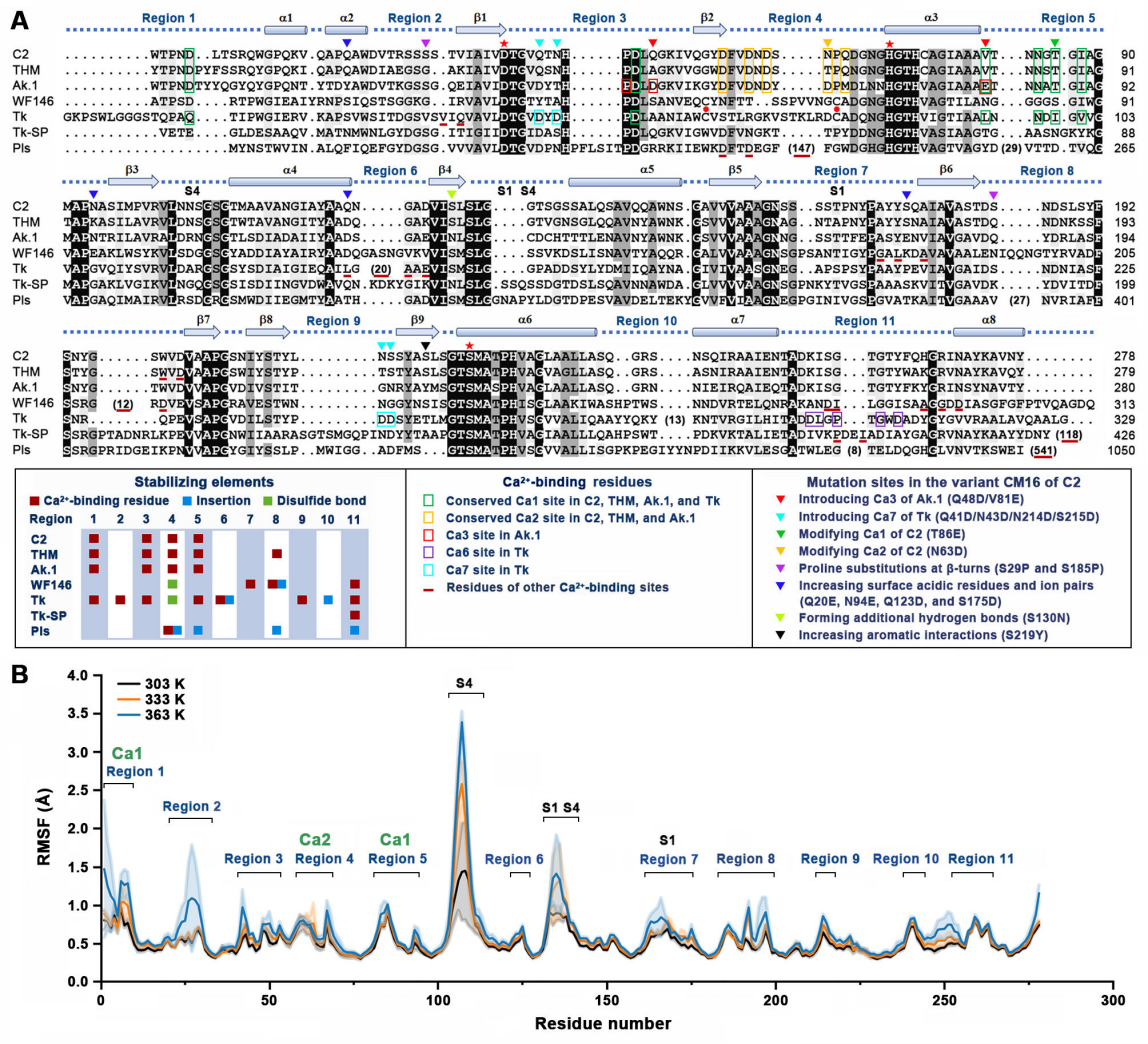
Stabilizing elements in protease C2 and its homologs. (**A**) Amino acid sequence alignment of protease C2 (ADD51544), thermitase (THM, 1105242A), protease Ak.1 (Q45670), protease WF146 (AAQ82911), Tk-subtilisin (P58502), Tk-SP (Q5JIZ5), and pyrolysin (Pls, P72186). The a-helices (a1–a8), b-strands (b1–b9), and the regions (regions 1–11) between or outside of the secondary structures are shown above the alignment. The catalytic residues are marked by red asterisks. The substrate-binding sites (S1 and S4) are indicated. The two Cys residues forming a disulfide bond in protease WF146 or Tk-subtilisin are indicated by red-filled circles. The number of amino acid residues of the insertion or C-terminal extension is shown in parentheses. Other symbols are explained in the box. (**B**) MD simulations of the Ca^2+^-bound form of protease C2 at different temperatures. The root-mean-square fluctuation (RMSF) values are expressed as means ± SDs (shaded area) of three independent 100-ns MD trajectories. The regions 1–11, the substrate-binding sites (S1 and S4), and the Ca^2+^-binding sites (Ca1 and Ca2) are indicated.

Protease C2 shares a high sequence identity with thermitase (76%) from *T. vulgaris* (54) and protease Ak.1 (62%) from *Bacillus* sp. Ak.1 (55) (Fig. 2A). By contrast, the three members of the thermitase family share low sequence identity with other (hyper)thermostable subtilases such as protease WF146 (56), Tk-subtilisin (57), Tk-SP (58), and pyrolysin (59), mainly because the latter four contain large insertions and/or long C-terminal extensions (Fig. 2A). The sequence alignment of these subtilases showed that some stabilizing elements such as Ca^2+^-binding sites, insertions, and disulfide bonds are located in the regions (regions 1–11) between or outside of the secondary structures (α-helix and β-strand) (Fig. 2A). It appears that the regions 1–11 represent structural hot spots for increasing thermostability in (hyper)thermostable subtilases. Moreover, Ca^2+^-binding sites could be detected in 10 out of the 11 regions of these enzymes (Fig. 2A), indicating that the binding of Ca^2+^ in these regions is a common strategy employed by subtilases to improve thermostability.

The MD simulations of protease C2 revealed relatively higher root-mean-square fluctuation (RMSF) values (the average of three independent 100 ns MD runs) in the regions 1–11 and the substrate-binding sites (S1 and S4), and the RMSF values increased with the increase in temperature (303–363 K) (Fig. 2B), suggesting that these regions are relatively flexible and thermal-sensitive. MD simulations were also performed for SM9 under the same conditions, showing that the RMSF values in some regions, particularly region 2 containing the stabilizing mutation S29P, were lower than those of protease C2 (Fig. S6). These results imply that rigidifying the flexible and thermal-sensitive regions could stabilize the enzyme.

### Empirical engineering of hyperthermostable variants by modifying and incorporating Ca^2+^-binding sites

A universal feature of subtilases is the presence of one or more Ca^2+^-binding sites (60). Protease C2 possesses two Ca^2+^-binding sites (Ca1 and Ca2), which are conserved in thermitase and protease AK.1, and the Ca1 site is also conserved in Tk-subtilisin (Fig. 2A). The Ca^2+^-binding residues of the Ca1 and Ca2 sites of protease C2 are located in the regions 1, 4, and 5, which show relatively higher RMSF values at higher temperatures (Fig. 2B), although the two sites are occupied by Ca^2+^ ions. We investigated whether the thermostability of protease C2 could be improved by modifying the Ca1 and Ca2 sites.

In the Ca1 site of protease C2, the Ca^2+^ ion is coordinated with the side chain oxygen atoms of Asp5 (O^δ2^), Asp46 (O^δ1^ and O^δ2^), and Asn84 (O^δ1^) and the main-chain carbonyl oxygen atoms from Val81, Thr86, and Ile88 (Fig. S7). In an attempt to improve enzyme stability by increasing Ca^2+^ affinity of the Ca1 site, we replaced Ca^2+^-binding residues (Val81 and Thr86) and adjacent Gly85 by negatively charged residues without eliminating the Ca^2+^-binding ligands, yielding the single-point variants V81D, V81E, G85D, G85E, T86D, and T86E. For comparison purposes, Gln/Asn substitutions were introduced into these sites to generate the variants V81N, V81Q, G85N, G85Q, T86N, and T86Q. Compared with protease C2, T86E displayed increased thermostability in the presence of 10 mM CaCl_2_, while all other variants showed decreased or similar levels of stability (Table 1). Given that T86E but not T86Q was more thermostable than protease C2, the introduced negatively charged side chain of Glu86 does act as a stabilizing factor in T86E.

In the Ca2 site of protease C2, the Ca^2+^ ion is coordinated with the side chain oxygen atoms of Asp56 (O^d2^), Asp59 (O^d1^), Asp61 (O^d1^ and O^d2^), and the main-chain carbonyl oxygen atom from Asn^63^ (Fig. S7). The variants D56N, D61N, and N63D, generated by automated computational design, did not show a significant change in thermostability compared to protease C2 (Table 1). We further performed mutational analysis on the Ca^2+^-binding residue Asp59. The variant D59N, in which the Ca^2+^-coordinating ligand of Asp59 (O^d1^) in protease C2 was replaced by that of Asn59 (O^d1^), showed a decreased thermostability (Table 1), implying the negatively charged side chain of Asp59 contributes to the stability of protease C2. In D59R and D59K, the corresponding Ca^2+^-coordinating interaction was disrupted, and the two variants tended to be autoproteolytically cleaved into two fragments (Fig. S2). We noticed that the autocleavage fragments of D59R and D59K are similar to those of subtilisin BPN’ (61) and protease WF146 (62), and the autocleavage sites of the latter two subtilases are both located within the region 4 (Fig. 2A). Although the modification of the Ca2 site did not generate a variant with improved stability, the results confirm that the Ca2 site confers the resistance of protease C2 to proteolytic cleavage at the region 4.

Compared with protease C2 and thermitase, protease Ak.1 possesses an additional Ca^2+^-binding site (Ca3) (55), of which the Ca^2+^-binding residues (Pro47, Asp50, and Glu81) are located within the regions 3 and 5 (Fig. 2A). The Ca3 site of protease Ak.1 was introduced into protease C2, generating the variant Ca3M (Q48D/V81E), with the Ca^2+^ ion being coordinated with the side chain oxygen atoms of Asp48 (O^d2^) and Glu81 (O^e1^ and O^e2^) and the main-chain carbonyl oxygen atom from P45 (Fig. S7). In the presence of 10 mM CaCl_2_, the variant Ca3M showed a significantly higher thermostability than protease C2 and a similar activity level to the latter at 60°C (Table 1), confirming the stabilizing effect of the introduced Ca3 site on the enzyme.

Tk-subtilisin contains seven Ca^2+^-binding sites, and the Ca6 and Ca7 sites contribute to the hyperthermostabilization of the enzyme (63). In Tk-subtilisin, the Ca^2+^-binding residues of the Ca7 site are located within the regions 3 and 9, while those of the Ca6 site reside in the region 11 (Fig. 2A). The Ca7 site of Tk-subtilisin was incorporated into protease C2 as the Ca4 site to construct the variant Ca4M (Q41D/N43D/N214D/S215D), with the Ca^2+^ ion being coordinated with the side chain oxygen atoms of Asp41 (O^d1^ and O^d2^), Asp43 (O^d1^), Asp214 (O^d1^), and Asp215 (O^d1^) (Fig. S7). The Ca6 site-containing region (Asp303 to Tyr313) of Tk-subtilisin was also incorporated into protease C2 as the Ca5 site to replace the corresponding region (Lys257 to His267) (Fig. 2A), generating the variant Ca5M (K257D/I258L/S259G/G260P/T263W/Y264D/F265A/Q266D/H267Y). Compared with protease C2, Ca4M showed an increased thermostability and a similar level of activity in the presence of 10 mM CaCl_2_ (Table 1). By contrast, Ca5M was almost completely inactivated after incubation at 85°C for 1 h (Table 1), probably because the sequence similarity of the region 11 and surrounding regions between protease C2 and Tk-subtilisin is very low (Fig. 2A), and the incorporated Ca5 site region may destabilize local structure.

**Fig 3:**
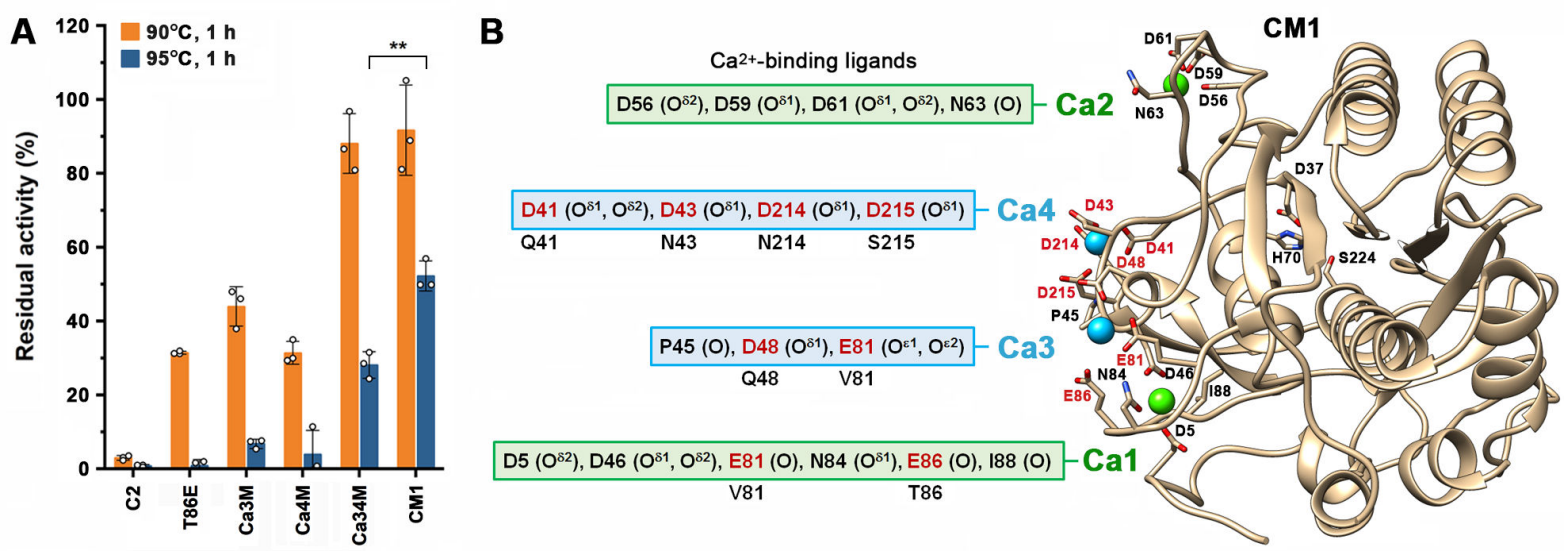
Thermostability of protease C2 and its variants with modified and/or introduced Ca^2+^-binding sites. (**A**) Thermal resistance of the enzymes. The enzymes (1 µg/mL) were incubated at 90°C or 95°C for 1 h in 50 mM Tris-HCl (pH 8) containing 10 mM CaCl_2_ and then subjected to azocaseinolytic activity assay at 60°C. The residual activity is expressed as a percentage of the original activity. The values are expressed as means ± SDs from three independent experiments (**P < 0.01; calculated by Student’s *t* test). (**B**) Structural model of CM1 predicted by AlphaFold 3. The Ca^2+^-binding ligands are shown, and the mutated residues were marked in red. The catalytic residues (D37, H70, and S224) are indicated.

The mutations in Ca3M and Ca4M were combined to construct the variant Ca34M. After incubation at 90°C for 1 h, Ca34M retained much higher residual activity (88.0%) than Ca3M (43.9%) or Ca4M (31.4%) (Fig. 3A). The stabilizing mutation T86E was then introduced into Ca34M to generate the variant CM1, which retained a significantly higher residual activity (49.8%) than Ca34M (26.4%) after incubation at 95°C for 1 h (Fig. 3A). In CM1, the Ca^2+^ ions in the Ca3 and Ca4 sites are coordinated with the ligands in the same way as in Ca3M and Ca4M (Fig. 3B; Fig. S7).

### A combination of stabilizing mutations generates variants with Ca^2+^-dependent boiling resistance

**Fig 4:**
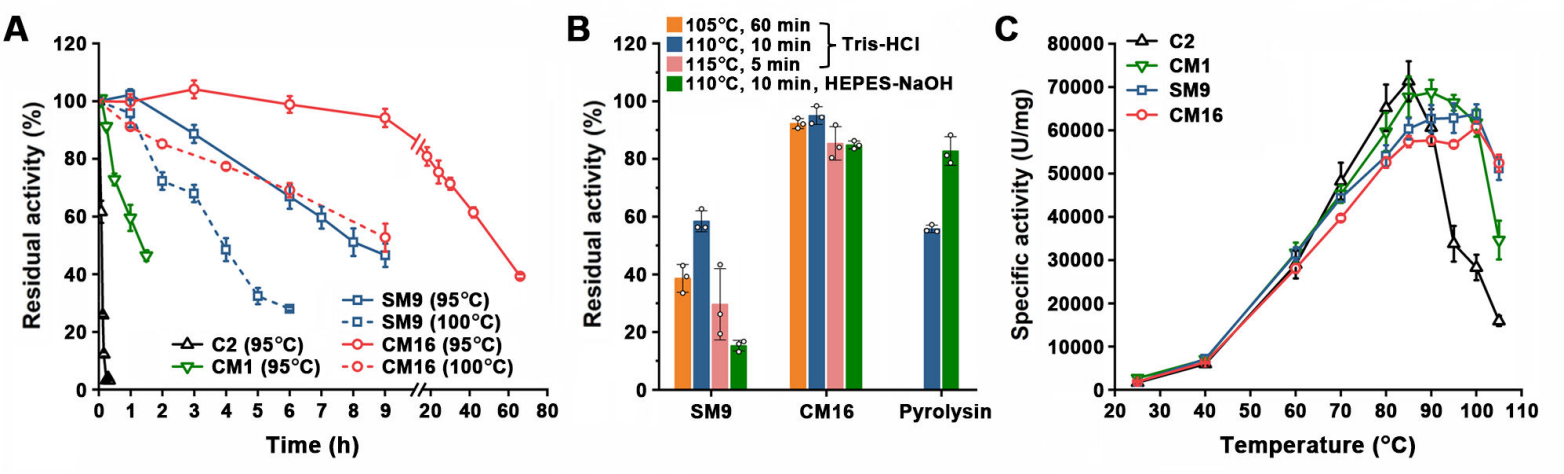
Thermostability and activity of the combination variants. (**A and B**) Thermal resistance of the enzymes. The enzymes (1 µg/mL) were incubated at the indicated temperatures for different time periods in 50 mM Tris-HCl (pH 8.0) or 50 mM HEPES-NaOH (pH 8.0) in the absence (pyrolysin) or presence (C2, SM9, and CM16) of 10 mM CaCl_2_ and then subjected to azocaseinolytic activity assay at 60°C (C2, SM9, and CM16) or 95°C (pyrolysin). The residual activity is expressed as a percentage of the original activity. (**C**) Temperature dependence of enzyme activity. The azocaseinolytic activity of the enzyme was determined at different temperatures. The values are expressed as means ± SDs from three independent experiments.

By sequential rounds of combination, the mutations in SM9 were introduced into CM1, generating a series of combination variants. In the presence of 10 mM CaCl_2_, these combination variants showed increased thermostability with the accumulation of the mutations, while their activity levels at 60°C remained similar to that of protease C2 (Table 2), reflecting the cumulative effect of the mutations on thermostability but not activity of the enzyme. CM1, SM9, and the final variant CM16 containing all of the 16 mutations in SM9 and CM1 exhibited half-lives of approximately 1.5 h, 8 h, and 50 h at 95°C, respectively, whereas protease C2 was almost completely inactivated after incubation at 95°C for 15 min (Fig. 4A). When incubated at 100°C, SM9 retained 48.6% residual activity after 4 h, while CM16 retained 52.7% residual activity after 9 h (Fig. 4A).

**TABLE 2:** Thermostabilities and activities of the combination variants^*a*^

Enzyme	Residual activity (%)		Relative activity (%)
	95°C, 9 h	100°C, 9 h	
C2	0.10 ± 0.10 (***)	ND	100.00 ± 12.49 (n.s.)
CM1	0.12 ± 0.09 (***)	ND	107.45 ± 5.84 (n.s.)
CM1 + S29P	3.03 ± 1.36 (***)	ND	88.62 ± 3.39 (*)
CM1 + N94E	1.86 ± 1.52 (***)	ND	99.12 ± 1.05 (n.s.)
CM1 + S185P	18.61 ± 8.72 (***)	ND	113.45 ± 1.31 (*)
CM1 + S219Y	1.84 ± 0.18 (***)	ND	102.77 ± 8.40 (n.s.)
CM1 + S130N	6.88 ± 2.40 (***)	ND	101.15 ± 4.18 (n.s.)
CM1 + S130 N/S29P	36.21 ± 8.63 (***)	ND	94.78 ± 1.10 (*)
CM1 + S130N/N94E	33.61 ± 2.17 (***)	ND	113.58 ± 4.60 (*)
CM1 + S130N/S185P	46.33 ± 7.07 (***)	ND	114.50 ± 0.62 (**)
CM1 + S130N/S219Y	31.35 ± 3.21 (***)	ND	99.59 ± 2.87 (n.s.)
CM1 + S130N/S29P/N94E	55.79 ± 3.58 (***)	ND	90.43 ± 1.19 (*)
CM1 + S130N/S29P/S219Y	52.54 ± 1.72 (***)	ND	97.82 ± 2.53 (n.s.)
CM1 + S130N/N94E/S219Y	57.22 ± 2.49 (***)	ND	85.79 ± 1.04 (**)
CM1 + S130N/S29P/S185P	63.66 ± 8.93 (**)	ND	92.97 ± 3.15 (n.s.)
CM1 + S130N/S219Y/S185P	66.99 ± 5.46 (**)	ND	116.18 ± 1.51 (**)
CM1 + S130N/N94E/S185P	74.45 ± 8.88 (*)	ND	94.80 ± 2.47 (n.s.)
CM1 + S130N/S29P/N94E/S219Y	71.64 ± 2.57 (***)	ND	91.14 ± 2.60 (*)
CM1 + S130N/S29P/N94E/S219Y/S185P	92.06 ± 5.69 (n.s.)	32.92 ± 2.19 (**)	99.42 ± 0.43 (n.s.)
CM16	94.14 ± 0.81	52.70 ± 4.86	101.97 ± 3.38

^*a*^ The enzymes (1 μg/mL) were incubated at 95°C or 100°C for 9 h in 50 mM Tris-HCl (pH 8) containing 10 mM CaCl_2_ and then subjected to azocaseinolytic activity assay at 60°C. The residual activity is expressed as a percentage of the original activity. The original activities of the enzymes were used to calculate the relative activity with the original activity of protease C2 defined as 100%. The values are expressed as means ± SDs from three independent experiments. Statistically significant differences between CM16 and other enzymes were calculated by Student’s *t* test (***P <0.001; **P < 0.01; *P < 0.05; n.s., no significance). ND, not determined.

The thermostabilities of SM9 and CM16 at temperatures above 100°C were investigated and compared with that of pyrolysin. Note that in contrast to SM9 and CM16, pyrolysin was analyzed in the absence of CaCl_2_ due to its sensitivity to metal ions, including Ca^2+^ (64). After heat treatment at 110°C for 10 min in 50 mM Tris-HCl (pH 8.0), SM9 and CM16 retained 58.1% and 94.6% residual activity, respectively, which are comparable to or higher than that (55.5%) of pyrolysin (Fig. 4B). When heat treated at 110°C for 10 min in 50 mM HEPES-NaOH (pH 8.0), SM9 retained lower residual activity (15.2%) than pyrolysin (82.3%), while CM16 showed comparable residual activity (84.4%) to pyrolysin (Fig. 4B). Compared with protease C2, the variants CM1, SM9, and CM16 displayed enhanced activities at and above 95°C, while SM9 and CM16 showed an optimum temperature of 100°C (Fig. 4C). These results confirm that protease C2 could be engineered to be boiling-resistant variants that are superior or at least comparable to hyperthermostable pyrolysin in terms of thermostability.

**Fig 5:**
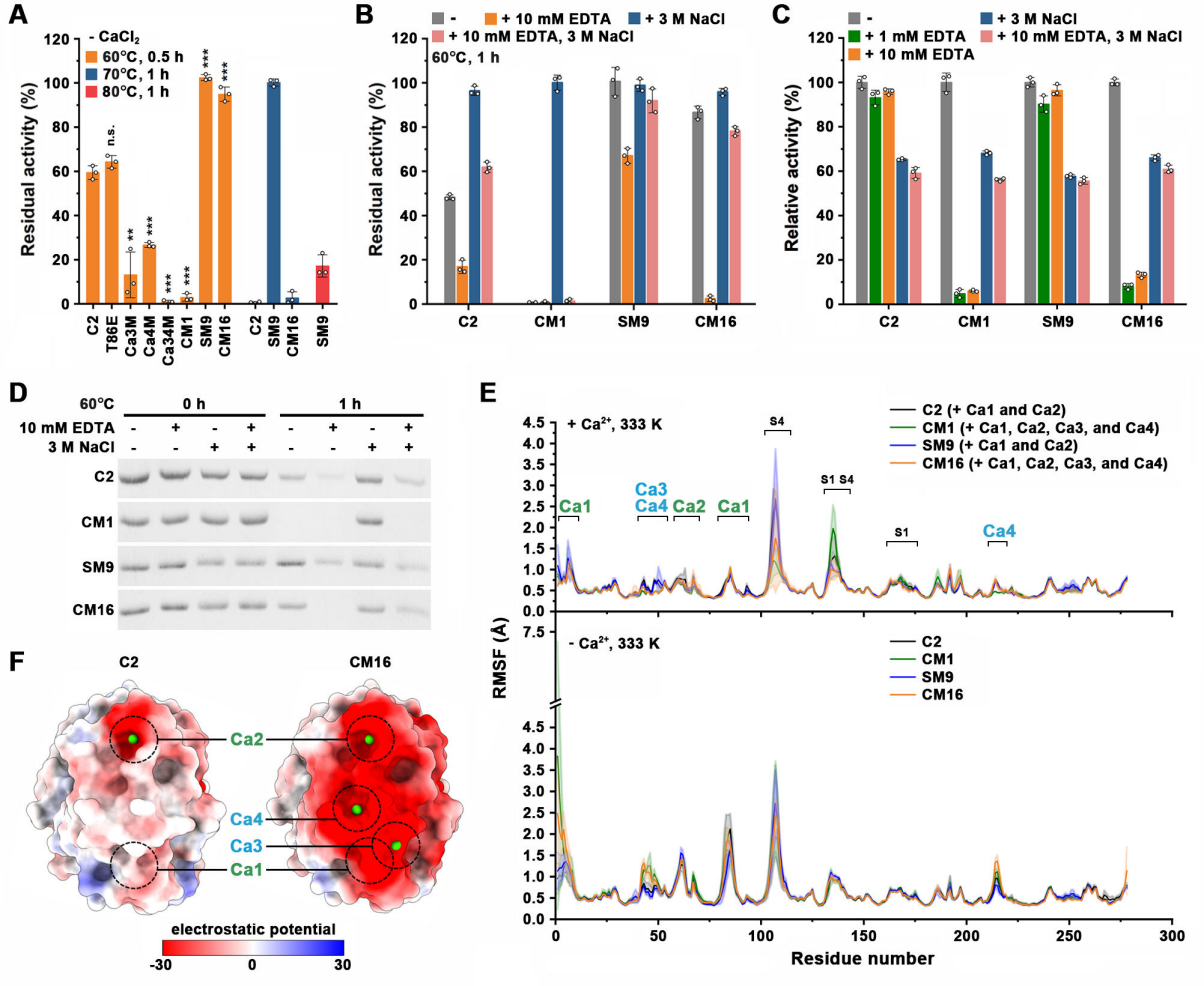
The Ca^2+^ dependence of protease C2 and its variants. (A, B, and D) Effects of EDTA and NaCl on enzyme thermostability. In the absence (–) of CaCl_2_, the enzymes (1 µg/mL) were incubated at the indicated temperatures for different time periods in 50 mM Tris-HCl (pH 8) without (–) or with (+) 10 mM EDTA and/or 3 M NaCl, followed by azocaseinolytic activity assay at 60°C (**A and B**) or SDS-PAGE analysis (**D**). The residual activity is expressed as a percentage of the original activity. (**C**) Effects of EDTA and NaCl on enzyme activity. The azocaseinolytic activity of the enzyme was determined at 60°C in 50 mM Tris-HCl (pH 8) without (–) or with (+) 1 mM or 10 mM EDTA and/or 3 M NaCl. The relative activity was calculated with the activity of the sample without the additive (–) defined as 100%. (**E**) MD simulations of the Ca^2+^-bound (+ Ca^2+^) and Ca^2+^-unbound (– Ca^2+^) forms of the enzymes at 333 K. The RMSF values are expressed as means ± SDs (shaded area) of three independent 100-ns MD trajectories for each enzyme. (**F**) The surface electrostatic potential of the enzyme. The Ca^2+^-binding sites (Ca1, Ca2, Ca3, and Ca4) are indicated. The values are expressed as means ± SDs from three independent experiments (***P < 0.001; **P < 0.01; *P < 0.05; n.s., no significance; calculated by Student’s *T* test) (A, B, and C).

We next investigated the Ca^2+^-dependence of the variants with modified and/or introduced Ca^2+^-binding sites. Protease C2 retained 54.4% activity after 1-h incubation at 85°C in the presence of 10 mM CaCl_2_ (Table 1), but lost more than 40% of its activity after incubation at 60°C for 0.5 h in the absence of CaCl_2_ (Fig. 5A), reflecting the Ca^2+^ dependence of the enzyme. The variant T86E showed similar thermostability to protease C2 in the absence of CaCl_2_ (Fig. 5A), although it is more thermostable than the latter in the presence of CaCl_2_ (Fig. 3A), reflecting the role of introduced Glu86 in stabilizing the enzyme via facilitating the binding of Ca^2+^ to the Ca1 site.

Although the variants Ca3M, Ca4M, Ca34M, and CM1 were much more thermostable than protease C2 in the presence of CaCl_2_ (Fig. 3A), they showed decreased thermostability in the absence of CaCl_2_ (Fig. 5A), suggesting that the introduced Ca3 and Ca4 sites stabilize the enzyme when bound to Ca^2+^ but destabilize the enzyme when not occupied by Ca^2+^. Moreover, in the presence of EDTA, the variant CM1 showed lower thermostability (Fig. 5B) and activity (Fig. 5C) than protease C2, and suffered from complete autodegradation after 1-h incubation at 60°C (Fig. 5D). These results reveal the high sensitivity of CM1 to EDTA and emphasize the destabilizing effects of the Ca^2+^-unbound Ca3 and Ca4 sites on the enzyme. By contrast, compared with protease C2, the variant SM9 lacking the Ca3 and Ca4 sites displayed increased thermostability in both the presence (Fig. 1B) and absence (Fig. 5A) of CaCl_2_, as well as enhanced resistance to EDTA (Fig. 5B, C and D). The variant CM16 combined the features of SM9 and CM1, showing increased thermostability in the absence of CaCl_2_ (Fig. 5A) and being more sensitive to EDTA than protease C2 (Fig. 5B, C and D).

We further performed MD simulations for the Ca^2+^-bound and Ca^2+^-unbound forms of protease C2, CM1, SM9, and CM16. The results showed that the RMSF values in the Ca^2+^-binding regions of their Ca^2+^-unbound forms were higher than those of their Ca^2+^-bound forms (Fig. 5E), confirming the stabilizing effect of Ca^2+^-binding on these enzymes. Particularly, compared with Ca^2+^-unbound protease C2 and SM9, the Ca^2+^-unbound CM1 and CM16 showed increased RMSF values in the Ca1, Ca3, and Ca4 sites (Fig. 5E) containing Asp/Glu substitutions (Fig. 3B), implying that the introduced acidic residues confer additional flexibility to these regions.

In protease C2, the surface electrostatic potential at the Ca1 and Ca2 sites is negative (Fig. 5F) because these regions are relatively rich in acidic residues (Fig. 3B). Compared with protease C2, the variant CM1 contains an additional eight acidic residues at the Ca1, Ca3, and Ca4 sites (Fig. 3B). As a combination variant of CM1 and SM9, CM16 contains both the additional eight acidic residues from CM1 and the additional four acidic residues from SM9 (Fig. 1A), which makes the surface electrostatic potential at the Ca1, Ca2, Ca3, and Ca4 sites highly negative (Fig. 5F) and leads to destabilization of the Ca^2+^-unbound enzyme by electrostatic repulsion.

In the absence of CaCl_2_, the supplementation of 3 M NaCl could increase the thermostability and decrease the autodegradation of protease C2, and the stabilizing effect of NaCl was more profound for CM1 (Fig. 5B and D), likely due to the weakening of electrostatic repulsion by increased ionic strength. NaCl also increased the EDTA resistance of protease C2, SM9, and CM16, in terms of their thermostability (Fig. 5B) and autodegradation sensitivity (Fig. 5D). Due to its high sensitivity to EDTA, CM1 was almost completely inactivated and degraded after EDTA treatment at 60°C for 1 h, even in the presence of 3 M NaCl (Fig. 5B and D). Nevertheless, the addition of 3 M NaCl into the reaction mixture containing EDTA could enhance the activity of CM1 (Fig. 5C). Most likely, in the reaction mixture, the protein substrates inhibited the severe autodegradation of CM1 in the presence of EDTA by reducing the chance of proteolytic interaction between enzyme molecules, allowing the enzyme to be stabilized by NaCl and to exhibit activity. We observed that in the absence of EDTA, the activity of protease C2, CM1, SM9, or CM16 decreased in the presence of 3 M NaCl (Fig. 5C), indicating an inhibitory effect of NaCl on enzymatic activity. Accordingly, the activity enhancement of CM1 and CM16 by NaCl in the presence of EDTA (Fig. 5C) is due to the stabilizing effect of NaCl on the enzyme, rather than salt-induced enzyme activation.

### The boiling-resistant variants are tolerant to polyextreme conditions

**Fig 6:**
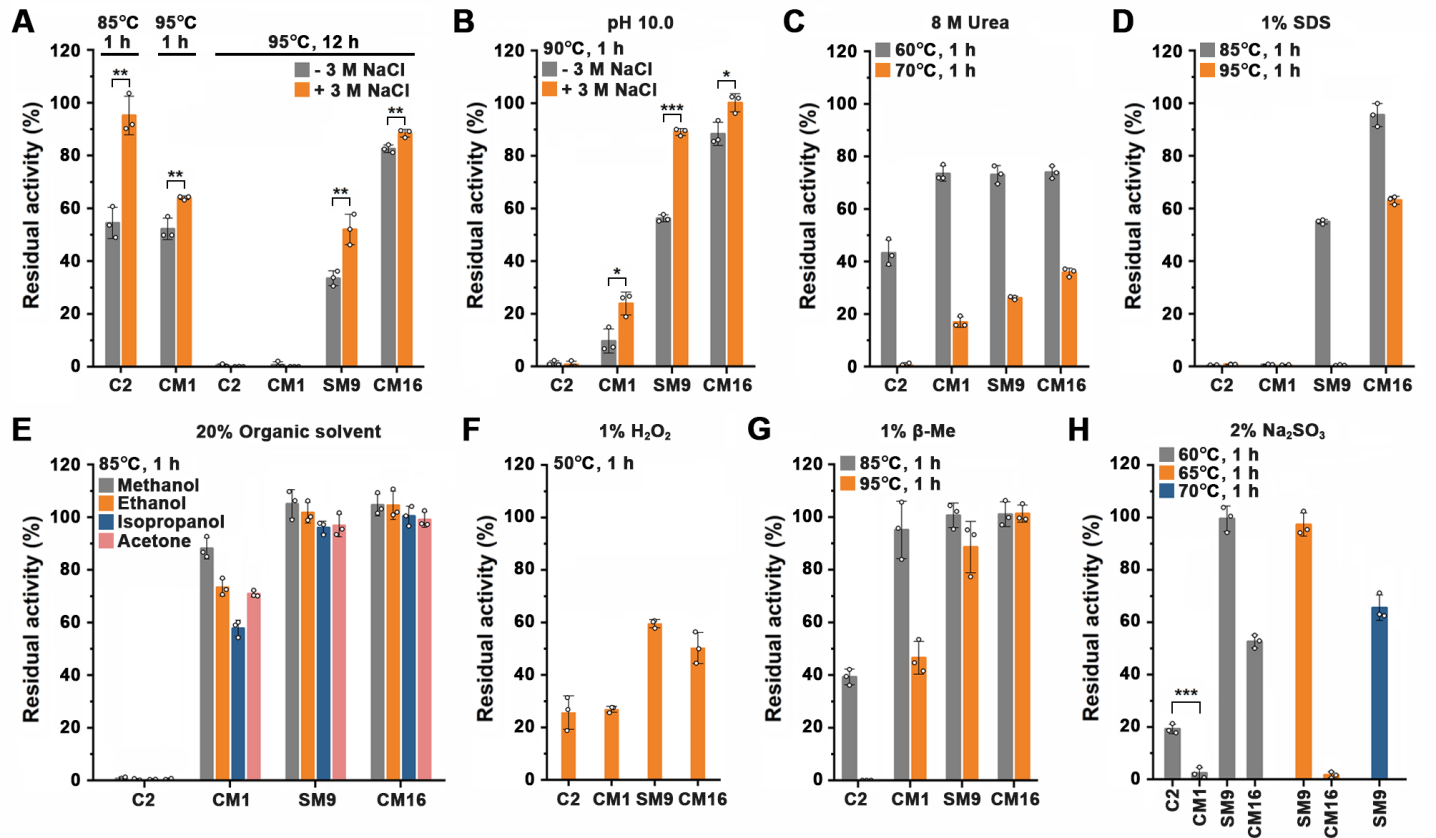
Resistance of protease C2 and its variants to extreme conditions. In the presence (**A–G**) or absence (**H**) of 10 mM CaCl_2_, the enzymes (1 µg/mL) were incubated at the indicated temperatures for different time periods in 50 mM Tris-HCl (pH 8) (**A, C–H**) or 50 mM Gly-NaOH (pH 10) (**B**) without (−) or with (+) different additives as indicated, followed by azocaseinolytic activity assay at 60°C. The residual activity is expressed as a percentage of the original activity. The values are expressed as means ± SDs from three independent experiments (***P < 0.001; **P < 0.01; *P < 0.05; calculated by Student’s *t* test).

The supplementation of 3 M NaCl could stabilize protease C2, CM1, SM9, and CM16 not only in the absence of CaCl_2_ or under chelating conditions (Fig. 5B) but also in the presence of CaCl_2_ (Fig. 6A) or under highly alkaline conditions (Fig. 6B), most likely by high ionic strength-induced attenuation of unfavorable electrostatic interactions and reinforcement of favorable hydrophobic interactions.

Besides the enhanced thermostability, the boiling-resistant SM9 and CM16 also showed increased stability at high pH value (pH 10) (Fig. 6B) or in the presence of 8 M urea (Fig. 6C), 1% SDS (Fig. 6D), 20% organic solvents (Fig. 6E), 1% H_2_O_2_ (Fig. 6F), or 1% b-Me (Fig. 6G) compared with protease C2. We noticed that in the absence of CaCl_2_, SM9 was highly resistant to 2% Na_2_SO_3_ compared with CM16, retaining 100.4%, 95.4%, and 63.0% residual activity after 1-h incubation at 60°C, 65°C, and 70°C, respectively (Fig. 6H), probably because the competition for Ca^2+^ between SO_3_^2−^ and the modified Ca1 site and the introduced Ca3 and Ca4 sites affects the stability of CM16.

### Degradation of native chicken feathers

**Fig 7:**
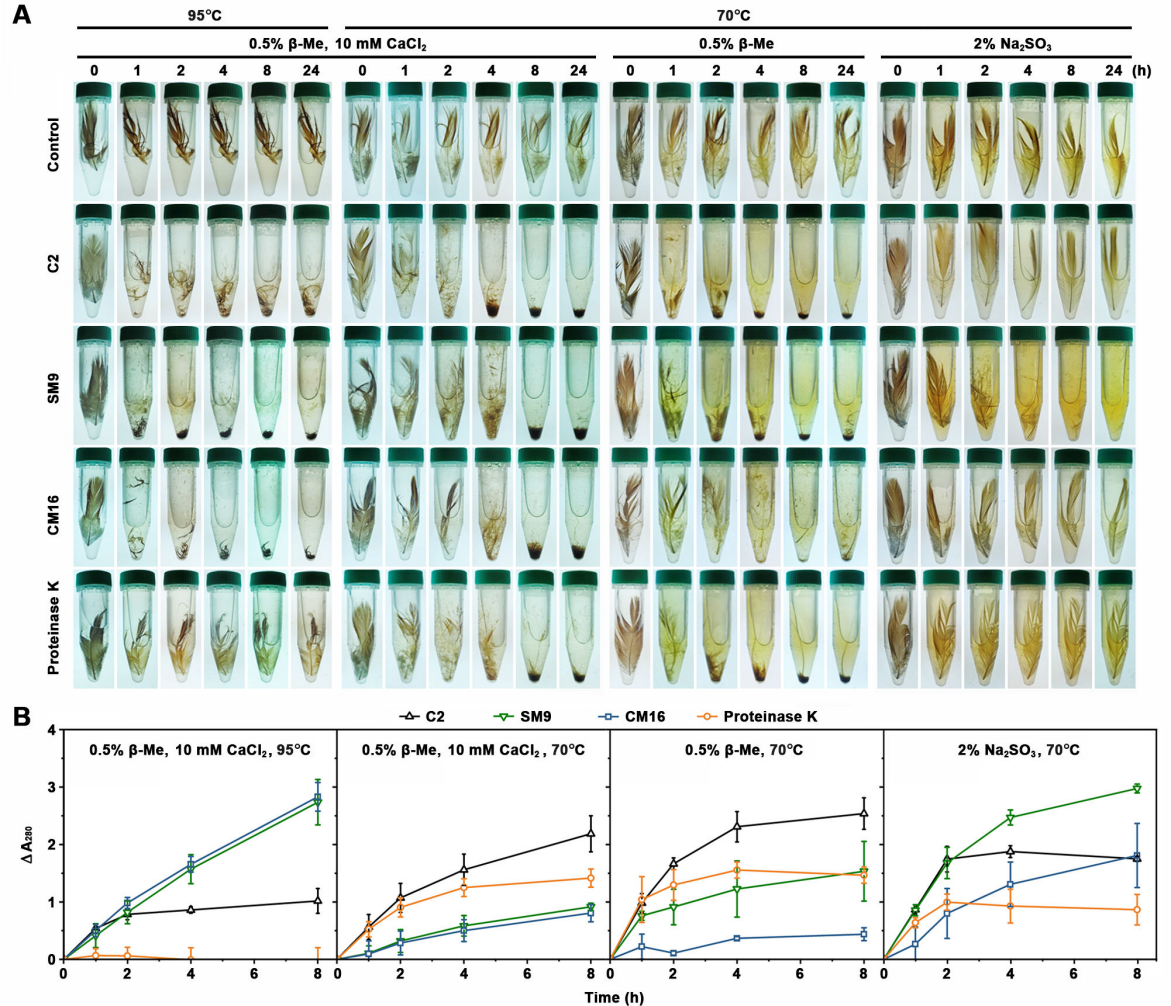
Comparison of the feather-degrading capacities of protease C2 and its variants. (**A**) Disintegration of native chicken feathers by the enzymes. The chicken feather (12 mg) was incubated at 70°C or 95°C in 5 mL of 50 mM Tris-HCl (pH 8) containing the indicated additive in the absence (control) or presence of the enzyme (25 µg/mL) for different time periods. (**B**) Soluble peptides released from feather meal by the enzymes. Feather meal (3 mg/mL) was incubated at 70°C or 95°C in 1.5 mL of 50 mM Tris-HCl (pH 8) containing the indicated additive in the absence (control) or presence of the enzyme (2 µg/mL). At different time points, the A_280_ value of the supernatant of the reaction mixture was measured, and the difference (ΔA_280_) between the A_280_ values of the experimental group and the control group was calculated. The values are expressed as means ± SDs from three independent experiments.

The feather-degrading capacities of protease C2 and its boiling-resistant variants SM9 and CM16 were investigated under various conditions, and proteinase K, well known for its keratinolytic activity (65), was used as a control. Considering the Ca^2+^ dependence of these enzymes, we first performed the feather-degrading reaction under reducing conditions (0.5% b-Me) in the presence of 10 mM CaCl_2_. By virtue of their high thermostability, SM9 and CM16 could completely disintegrate native feather within 4 h at 95°C; under the same conditions, protease C2 only partially disintegrated feather and proteinase K was unable to degrade the substrate due to thermal inactivation (Fig. 7A). Consistent with this, SM9 and CM16 displayed a higher keratinolytic activity than protease C2 and proteinase K at 95°C, as evidenced by the finding that a larger amount of soluble peptides, evaluated based on the absorbance at 280 nm (A280), was released from insoluble feather meal by SM9 and CM16 than by protease C2 at this temperature (Fig. 7B). When the reaction was performed at 70°C, all of the four enzymes could completely disintegrate native chicken feather within 24 h (Fig. 7A). In contrast to the situation at 95°C, protease C2 exhibited a higher hydrolytic activity against feather meal than SM9 and CM16 at 70°C (Fig. 7B), implying the mutations in the two variants affect the keratinolytic activity of the enzyme.

Given the difference in the Ca^2+^ dependence of thermostability among the enzymes, the feather-degrading reaction was also carried out without the addition of CaCl_2_. In the presence of 0.5% b-Me and at 70°C, protease C2, SM9, and proteinase K could completely disintegrate native feather; by contrast, CM16 only partially disintegrated the feather (Fig. 7A), reflecting its high Ca^2+^ dependence for thermostability. We observed that for the reactions catalyzed by protease C2, SM9, and proteinase K at 70°C in the presence of 0.5% b-Me, the amount of insoluble disintegration products (deposited at the bottom of the tube) of feather in the reaction mixture with 10 mM CaCl_2_ was higher than that without CaCl_2_ (Fig. 7A). Under the corresponding conditions, the amount of soluble peptides released from feather meal by the three enzymes in the presence of 10 mM CaCl_2_ was lower than that in the absence of CaCl_2_ (Fig. 7B). We found that the soluble degradation products of feather tended to be precipitated by CaCl_2_ (Fig. S8), which may account for the observed lower feather-degrading activity of the enzymes in the presence of 10 mM CaCl_2_.

When 2% Na_2_SO_3_ instead of 0.5% b-Me was used for feather-degrading reaction at 70°C, complete disintegration of native feather was achieved only for SM9 within 24 h (Fig. 7A), and the soluble degradation products released from feather meal by SM9 were higher than that by protease C2, CM16, or proteinase K (Fig. 7B), most likely due to the higher resistance of SM16 to Na_2_SO_3_ compared with the other enzymes (Fig. 6H).

**Fig 8:**
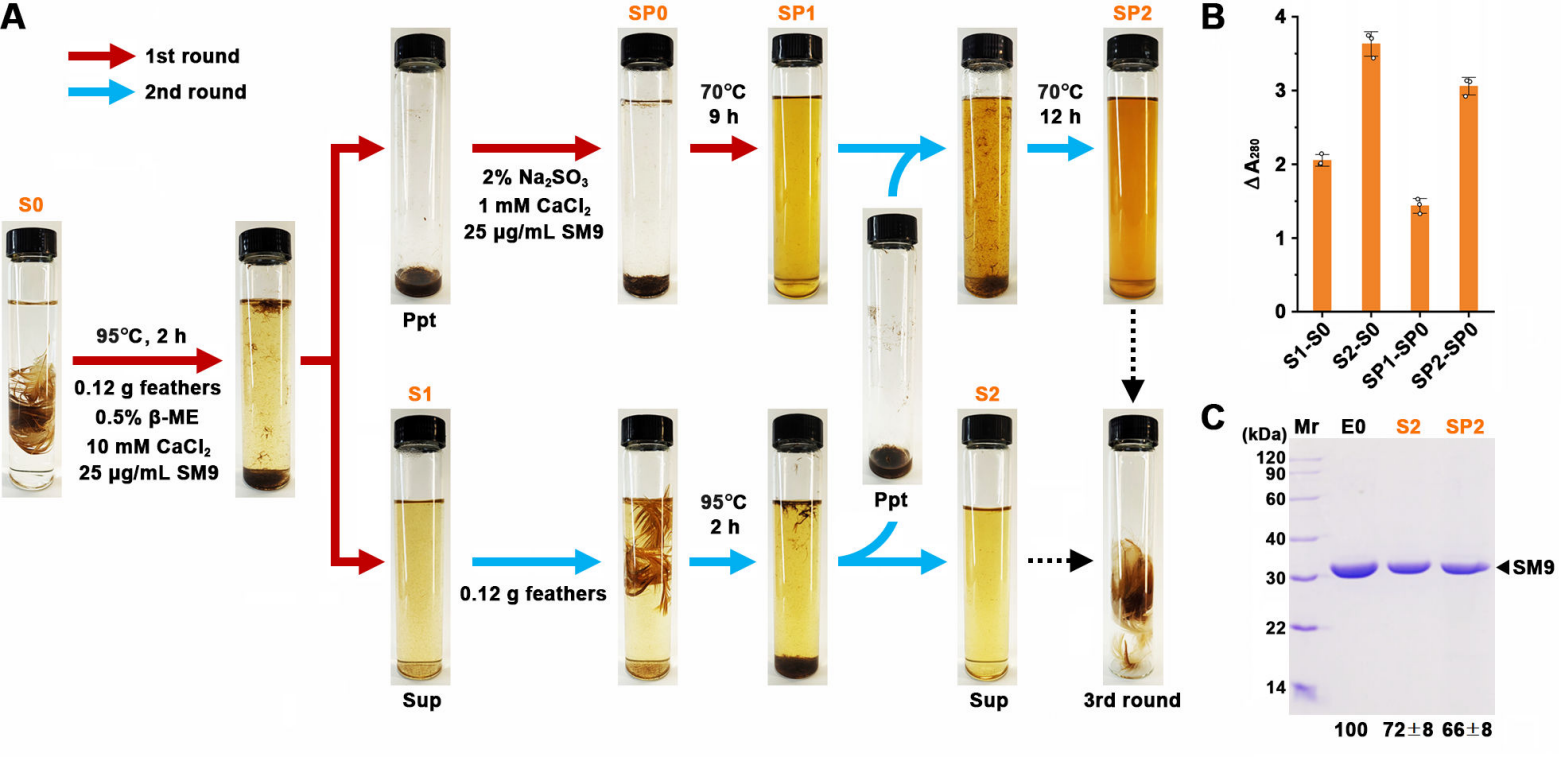
Successive rounds of degradation of native chicken feather by SM9 via enzyme recycling. The reactions were carried out at the indicated conditions in 50 mL of 50 mM Tris-HCl (pH 8) containing the indicated additives, and the supernatant (Sup) and pellet (Ppt) of the reaction mixture were separated by centrifugation at 13,400 × *g* for 10 min (**A**). The A_280_ values of the soluble fractions of the samples (S0, S1, S2, SP0, SP1, and SP2) were measured for the calculation of the increment of the A_280_ value (ΔA_280_) (**B**). The initial enzyme sample (E0) and the samples S2 and SP2 of the second-round reaction mixture were subjected to SDS-PAGE analysis, and the densitometric ratios of the band of SM9 in S2 and SP2 compared with that of E0 are shown below the gel (**C**). The values are expressed as means ± SDs from three independent experiments. The third round of degradation is shown in Fig. S9.

Based on the above evidence, we developed a procedure consisting of successive rounds of degradation of native chicken feather via enzyme recycling, involving the rapid disintegration of feather by SM9 within 2 h at 95°C in the presence of 0.5% β-Me and 10 mM CaCl_2_, the complete enzymatic degradation of the insoluble disintegration products (Ppt) into soluble peptides at 70°C in the presence of 2% Na_2_SO_3_ and 1 mM CaCl_2_, and reusing the enzyme-containing soluble fraction (Sup) of degradation products to disintegrate newly added feather at 95°C (Fig. 8A). After two rounds of degradation by SM9, a total of 0.24 g feathers were completely converted into soluble products (Fig. 8A), and the amount of soluble peptides released from feather increased with the increase in degradation reaction rounds (Fig. 8B). SM9 was highly stable during this process, approximately 72% or 66% of the enzyme molecules were retained after two rounds of reaction at 95°C for 4 h (sample S2) or at 70°C for 21 h (sample SP2) (Fig. 8C), allowing a third round of complete degradation of additional 0.12 g of feather (Fig. S9).

## DISCUSSION

### Computational design and empirical engineering

In this study, we employed automated computational tools, a structure-based rational approach, and empirical design to further improve the thermostability of protease C2, a thermostable keratinolytic subtilase. The combination of the stabilizing mutations yields boiling-resistant variants such as SM9 and CM16 with hyperthermostability superior to or at least comparable to pyrolysin (12), one of the most thermostable proteases known to date. To the best of our knowledge, this is the first report on the engineering of a subtilase with boiling resistance.

Among the 35 single-point variants with a stabilizing mutation predicted by automated computational tools, only four (Q20E, S29P, S130N, and S219Y) were experimentally verified to be significantly more thermostable than protease C2. This is consistent with previous findings that a large fraction of stabilizing point mutations predicted by computational design tools appear to be neutral or destabilizing when tested experimentally, probably because the amount of existing experimental data for training deep-learning models is too limited to allow deeper algorithms (46, 66). In particular, protease C2 itself is a thermostable enzyme, and it is highly likely that available experimental data from more thermostable enzymes for deeper algorithms is very limited. Supporting evidence comes from the findings that none of the selected mutations predicted by ThermoMPNN were experimentally confirmed to display increased thermostability. Additionally, although HyperMPNN is a deep-learning tool derived from ProteinMPNN by training on proteins from hyperthermophiles (37), only three (Q20E, S29P, and S130N) of the 17 mutations predicted by this tool showed significantly higher thermostability than protease C2. With the rapid accumulation of information on proteins from hyperthermophiles in the database, the prediction accuracy for thermostabilizing mutations by computational tools is expected to improve largely (67).

Although most of the predicted stabilizing mutations predicted by the computational tools were experimentally characterized to be neutral or destabilizing, we cannot exclude the possibility that these mutations could stabilize the enzyme when combined. This is supported by the evidence that the mutations N63D, Q123D, and S175D could synergistically or cumulatively improve the thermostability of SM4. Nevertheless, the variant FP7 with multiple-point mutations designed by FireProt 2.0 showed decreased rather than increased thermostability, probably due to current limitations of the databases for constructing multiple-sequence alignment to estimate the conservation coefficient of each residue and the force-field-based method itself (35).

By structure-based rational design, Asp/Glu substitutions were individually introduced into protease C2 to form additional ion pairs. The experimental data reveal that the Asp/Glu substitution at position 94 (N94E and N94D) but not position 175 (S175D and S175E) resulted in a significant increase in thermostability, suggesting that the effect of the introduced ion pair on enzyme stability depends on its location on the enzyme. Among the five rationally designed variants with Pro substitutions at the b-turns, only S185P was experimentally verified to be more thermostable than protease C2, which also emphasizes the importance of the location of introduced proline for stabilizing the enzyme.

In terms of empirical design, we focused on Ca^2+^-binding sites, which are well known to play important roles in stabilizing subtilases (6, 60). It has been reported that the incorporation of the Ca2 site of thermitase into mesophilic subtilisin BPN′ generates a variant (Mut 2) with improved thermostability at 60°C in the presence of CaCl_2_ (61). Similarly, the incorporation of the Ca3 site of protease Ak.1 and the Ca7 site of Tk-subtilisin into protease C2 greatly stabilizes the resulting variant Ca34M near the boiling point in the presence of CaCl_2_. By contrast, the incorporation of the Ca6 site of Tk-subtilisin into protease C2 destabilizes rather than stabilizes the enzyme, implying that the incorporated Ca6 site affects local stabilizing forces. In this context, the incorporation of Ca^2+^-binding sites is a promising strategy to engineer highly thermostable subtilases, on the premise that the incorporated element is compatible with the corresponding local structural environment.

The boiling-resistant variants, such as SM9 and CM16, were obtained by a combination of the stabilizing mutations, suggesting the cumulative effect of these mutations on the enzyme. In this study, however, only a small portion of the predicted stabilizing mutations by automated computational tools was experimentally tested, and limited stabilizing factors (e.g., ion pair, Pro substitution, and Ca^2+^-binding site) were considered for rational design and empirical engineering. Further validation of the predicted mutations in combination with rationally and empirically optimizing other stabilizing interactions is expected to identify additional stabilizing mutations for engineering of more thermostable variants.

### Stabilizing mechanism

**Fig 9:**
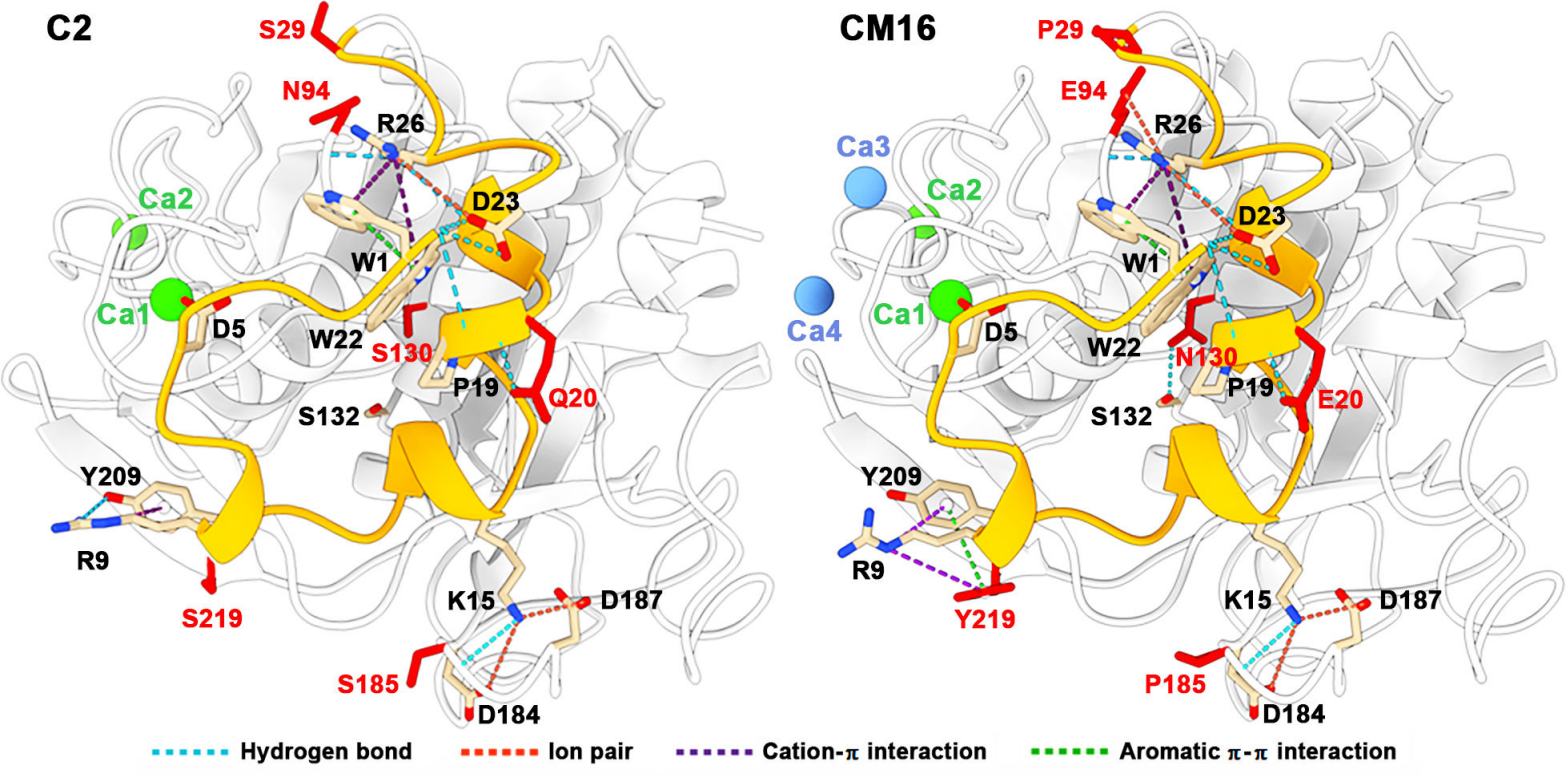
The effects of the mutations on enzyme structure. The residues before and after mutation are marked in red in protease C2 and CM16, respectively. The N-terminal region (the residues Trp1 to Ser29 or Pro29) is shown in orange. Dotted lines represent different interactions as indicated.

Compared with protease C2, the boiling-resistant CM16 contains 16 amino acid substitutions, combining the mutations in SM9 (Q20E, S29P, N63D, N94E, Q123D, S130N, S175D, S185P, and S219Y) and CM1 (Q41D, N43D, Q48D, V81E, T86E, N214D, and S215D). The boiling resistance of CM16 is achieved by the cumulative effects of various stabilizing forces conferred by these mutations. Among the mutations coming from SM9, S29P, and S185P rigidify the b-turns, while N94E introduces a long-range ion pair (Glu94-Arg26) to form an ionic network (Asp23-Arg26-Glu94, Fig. 9). The mutation S219Y introduces an aromatic π-π interaction (Tyr209-Tyr219) and a cation-π interaction (Arg9-Tyr219, Fig. 9). S130N leads to the formation of an extra hydrogen bond (Asn130-Ser132, Fig. 9). Similarly, the same mutation (S136N) at the corresponding position of protease WF146 also thermally stabilizes the enzyme by forming additional hydrogen bonds (68). For the mutations coming from CM1, T86E introduces a negative charge to improve the Ca^2+^ affinity of the Ca1 site, while those of the incorporated Ca3 and Ca4 sites stabilize the enzyme by binding Ca^2+^ to reduce the local flexibility, similar to the case of subtilisin BPN′ (61).

Importantly, the majority of the mutations in CM16 are involved in stabilizing the N-terminus region of the enzyme. In protease C2, the residue Trp1 is anchored to the enzyme surface by forming hydrogen bonds with Pro19 and Asp23, an aromatic π-π interaction with Trp22, and a cation-π interaction with Arg26; meanwhile, Asp5 binds to Ca^2+^ in the Ca1 site and Arg9 forms a hydrogen bond and a cation-π interaction with Tyr209 (Fig. 9). The decrease in the thermostability of the single-point variants K15A and R26A reveals the roles of Lys15 and Arg26 in stabilizing the N-terminal region of protease C2. The residue Lys15 forms a long-range ion pair with Asp184, a salt bridge with Asp187, and a hydrogen bond with Ser185, while Arg26 forms cation-π interactions with Trp1 and Trp22, hydrogen bonds with Trp22 and Asn94, and an ion pair with Asp23 (Fig. 9). In the variant CM16, the stabilizing interactions around the N-terminal region are reinforced by rigidifying the b-turns (S29P and S185P) to stabilize the interactions of Arg26, Asp184, and Asp187 (within or neighboring the b-turn) with their ligands (Lys15, Trp22, Asp23, and Asn94), forming the ionic network (Asp23-Arg26-Glu94, N94E) and the cation-π interaction (Arg9-Tyr219, S219Y), and strengthening the interactions between Ca^2+^ and its coordinating residues including Asp5 in the modified Ca1 site (Fig. 9).

The mutation Q20E does not bring a remarkable change in enzyme structure, wherein the side chain of either the substituted residue Glu20 in CM16 or the original residue Gln20 in protease C2 only forms a hydrogen bond with its own main-chain carbonyl oxygen (Fig. 9). Nevertheless, because the single-point variant Q20E shows increased thermostability, it is evident that the introduction of negatively charged Glu20 stabilizes the enzyme by influencing local electrostatic environment, although the exact underlying mechanism remains to be elucidated. The main-chain carbonyl oxygen of Asn63 acts as a Ca^2+^-coordinating ligand in the Ca2 site of protease C2, and the introduction of a negative charge by the mutation N63D may improve the Ca^2+^ affinity of the Ca2 site. The mutation Q123D leads to the formation of a long-range ion pair (Arg9-Asp46) in the single-point variant Q123D but not in CM16 (Table S1), and may confer stability to the enzyme by influencing local electrostatic environment in a manner similar to Q20E. The mutation S175D introduces a long-range ion pair (Asp175-Arg248) to form an ionic network (Asp175-Asp200-Arg248-Glu252, Table S1). Although the stabilization effects of the three individual Asp substitutions (N63D, Q123D, and S175D) appear to be too small to cause a significant increase in thermostability of the enzyme, they may cumulatively or synergistically improve the enzyme stability.

The mutations from CM1 for modifying and incorporating the Ca^2+^-binding sites are all negatively charged residues, making the surface electrostatic potential around the Ca^2+^-binding sites of CM16 highly negative. When these Ca^2+^-binding sites are not occupied by Ca^2+^, the electrostatic repulsion between negatively charged residues would destabilize the enzyme, and thus CM16 with more acidic residues is more sensitive to EDTA treatment than protease C2. Because the electrostatic repulsion would be weakened with the increase in ionic strength of the solvent, it is reasonable that the presence of 3 M NaCl could stabilize the enzyme.

### Application potential

By virtue of their hyperthermostability, the boiling-resistant variants SM9 and CM16 could rapidly degrade chicken feathers at 95°C under reducing conditions. So far, there is no published report on the engineering of keratinase with boiling resistance, and only a few naturally occurring keratinases capable of efficiently degrading keratin at temperatures around the boiling point have been reported. For instance, a membrane-bound serine protease (FIKase) of *Fervidobacterium islandicum* AW-1 exhibits optimal activity toward soluble keratin at 100°C and shows strong keratinolytic activity against native feathers at 90°C in the presence of dithiothreitol but not in the absence of the reducing agent (69), although the gene encoding FIKase remains unclear to date. The subtilisin-like keratinase GacK from *G. acetivorans* displays an optimum temperature of 100°C to degrade insoluble keratin azure in the absence of a reducing agent (19). Because purified keratinases are generally unable to degrade keratin (approximately 10% degradation reported in some cases) in the absence of reducing agents (9), it remains to be determined whether the presence of reducing agents could improve the keratinolytic activity and influence the temperature dependency of GacK. Keratinases have been used for degrading keratin wastes (e.g., feather and wool) into digestible peptides and amino acids to prepare nutritionally rich animal feed (9). The application of boiling-resistant keratinases in the feed industry has the advantage of simplifying the procedure for the production of keratin-based feed products by combining the enzymatic keratinolysis process and the disinfection process together at temperatures near or at the boiling point.

Hyperthermostable subtilases could efficiently degrade pathogenic proteins, such as prions at high temperatures, not only showing great potential application in the food and feed industry to prevent prion transmission but also meeting the recommended temperature requirements (80°C–95°C) for disinfection of medical instruments by washer-disinfectors (ISO 15883) (21, 24). Nevertheless, in certain circumstances, the hyperthermostability may be disadvantageous for applications of the enzymes. For instance, despite complete digestion of infectious prion proteins, pyrolysin is difficult to thermally inactivate, which may limit its applications in the medical field (22). Here, we found that the highly Ca^2+^-dependent CM16 is hyperthermostable in the presence of Ca^2+^ but is unstable in the absence of Ca^2+^ or in the presence of EDTA or Na_2_SO_3_. The high Ca^2+^ dependence of CM16 provides a simple and effective strategy to modulate the stability and activity of the enzyme by regulating Ca^2+^ concentration or adding Ca^2+^ chelators, which will expand the application field of the enzyme. Moreover, the hyperthermostable variants SM9 and CM16 are also highly resistant to high pH, high salinity, urea, SDS, and organic solvents. Such properties would be advantageous for them to find widespread use in many areas.

## MATERIALS AND METHODS

### Materials, strains, and chemicals

Restriction enzymes were purchased from Thermo Fisher Scientific (Waltham, MA, USA). DNA polymerase 2× KeyPo Master Mix (Dye Plus) was purchased from Vazyme (Nanjing, China). Proteinase K and azocasein were from Sigma (St. Louis, MO, USA). Isopropyl-β-D-thiogalactopyranoside (IPTG), kanamycin sulfate, and bovine serum albumin (BSA) were from Solarbio (Beijing, China). Pyrolysin was prepared as described previously (64). The plasmid pET26b was from Novagen (Darmstadt, Germany) and used as the expression vector. *E. coli* DH5α and *E. coli* BL21(DE3) were used as hosts for cloning and protein expression, respectively. All other chemical reagents are analytically pure.

### Plasmid construction and mutagenesis

The primers used for PCR are listed in Table S3. The gene encoding the signal peptide-lacking proform of protease C2 was amplified from previously constructed plasmid pET26b-C2 harboring the gene encoding protease C2 precursor (39) and inserted into the *Nde*I-*Eco*RI site of pET26b to construct the expression plasmid pET26b-pro-C2 for protease C2 proform with a C-terminal His-tag. The QuikChange site-directed mutagenesis method (70) was employed to introduce point mutations into target proteins. To construct the expression vector for the variant Ca5M, the 5′ and 3′ regions of the gene were amplified from pET26b-pro-C2 and subcloned into pET26b using Ready-To-Use Seamless Cloning Kit (Sangon Biotech, Shanghai, China). All recombinant plasmids were confirmed by DNA sequencing.

### Expression and purification

Recombinant proforms of the enzymes were produced in *E. coli* BL21 (DE3) as described previously (42). The cells were harvested and suspended in 50 mM Tris-HCl (pH 8.0) containing 10 mM CaCl_2_, sonicated on ice, and then centrifuged at 13,400 × *g* for 10 min at 4°C. The resulting supernatants containing the proforms were incubated at 80°C for 15 min to activate the enzymes*,* followed by centrifugation at 13,400 × *g* for 10 min to separate the soluble and insoluble fractions. The mature enzymes with a C-terminal His-tag in the soluble fraction were purified using affinity chromatography on a Ni^2+^-charged Chelating Sepharose Fast Flow resin column (GE Healthcare) as described previously (61). The elution fractions containing purified enzymes were dialyzed against 50 mM Tris-HCl (pH 8.0) or 50 mM HEPES-NaOH (pH 8.0) in the absence or presence of 10 mM CaCl_2_ overnight at 4°C. The purity of the enzyme was examined by SDS-PAGE analysis. The concentrations of purified enzyme samples were determined using the Bradford assay method with BSA as the standard.

### SDS-PAGE

SDS-PAGE was performed using a Tris-glycine or Tris-tricine buffer system. To prevent autodegradation of the protease during sample preparation (boiling) or electrophoresis, proteins were acid denatured and precipitated with 20% (wt/vol) trichloroacetic acid (TCA), washed with ice-cold acetone before being subjected to SDS-PAGE analysis.

### Proteolytic activity assay

Unless otherwise indicated, the azocaseinolytic activity of the enzyme was carried out at 60°C for 10 min in 300 µL of reaction mixture containing 150 µL of properly diluted enzyme sample and 0.25% (wt/vol) azocasein in 50 mM Tris-HCl (pH 8.0) containing 10 mM CaCl_2_. The reaction was terminated by the addition of 300 µL of 40% TCA. After standing at room temperature (~25°C) for 15 min, the mixture was centrifuged at 13,400 × *g* for 10 min, and the absorbance of the supernatant at 335 nm (*A_335_*) was measured in a 1 cm light-path cell. One unit (U) of azocaseinolytic activity was defined as the amount of enzyme required to increase the *A_335_* value by 0.01 unit per minute under the assay conditions.

### Enzymatic degradation of chicken feathers and feather meal

Chicken feathers obtained from a local farm were washed with tap water, soaked in 70% ethanol for 1 h, rinsed with deionized water, and dried. The enzymatic degradation of chicken feathers was conducted as described previously (43) with some modifications. Briefly, the cleaned feather was incubated with the enzyme under the conditions specifically indicated elsewhere. At different time intervals, the disintegration of the feather was recorded photographically.

To prepare feather meal, the cleaned feathers were cut into pieces about 2–3 mm in length, ground into a fine powder, and sieved through a 40-mesh sieve. Feather meal was incubated with the enzyme under the conditions specifically indicated elsewhere. At different time intervals, the reaction mixture was centrifuged at 13,400 × *g* for 10 min to collect the supernatant. The amount of soluble peptides released from feather meal was quantified by measuring the absorbance of the supernatant at 280 nm in a 1 cm light-path cell.

### Bioinformatic analyses

All of the structural models of protease C2 and its variants were predicted by AlphaFold 3 (https://alphafoldserver.com/) (33). The UCSF Chimera (71) and UCSF ChimeraX (72) were used for visualization of the structural model and the analysis of hydrogen bonds, cation-π interactions, and aromatic π-π interactions. Ion pairs were analyzed using the “Salt bridges” plugin (version 1.1) (https://www.ks.uiuc.edu/Research/vmd/plugins/saltbr/) in the VMD software (49), and the distance limits were chosen to be 4.0 Å and 8.0 Å to find salt bridges (50) and long-range ion pairs (48), respectively. The structural model of the enzyme was submitted to the PDBsum server (https://www.ebi.ac.uk/thornton-srv/databases/pdbsum) (73), where secondary structure annotations were generated using PROMOTIF (74), enabling the identification of the type I β-turns. The RASA of the amino acid residue, which is defined as the ratio of side-chain surface area to “random coil” value per residue, was calculated using GetArea (http://curie.utmb.edu/getarea.html) (51) with a probe radius of 1.4 Å. The RASA of an ion pair is the average of the RASA values of the two residues forming the ion pair (52). The residues or ion pairs with RASA value ≥20% are considered to be exposed to the solvent (52). The calculation of protein electrostatic surfaces was performed by APBS (75).

### Automated computational design

The structural model of the Ca^2+^-bound (Ca1 and Ca2 sites) form of protease C2 was submitted to the servers of the automated computational tools for the design of stabilizing mutations.

For the designs by FireProt 2.0 (https://loschmidt.chemi.muni.cz/fireprot/) (35), default energy thresholds for FoldX and Rosetta were applied, and default frequency thresholds for consensus analysis were applied. Predictions for stabilizing single-point mutants were based on either energy or consensus analysis. All single-point mutations were compiled into a complete protein sequence (FireProt-single). The energy- and evolution-based approaches were combined to predict stabilizing multiple-point mutations with smart filtering. The low-risk combination (FireProt-LRC) mutant was used for constructing the multiple-point variant FP7.

For the designs by PROSS 2 (https://pross.weizmann.ac.il/step/pross-terms/) (34), multiple sequence alignment parameters were set to default, and ref2015 was selected for the energy function. Nine variant sequences were generated, and Design 9, containing the maximum number of mutations, was used for subsequent sequence analysis.

For the designs by ProteinMPNN (https://app.tamarind.bio/tools/proteinmpnn) (36) and HyperMPNN (https://app.tamarind.bio/tools/hypermpnn) (37), the catalytic triad of protease C2 was fixed to ensure the catalytic function, and sequence optimization was performed at other positions, while explicitly excluding Cys substitutions. With noise set to 0.2, 50 sequences were generated at each of three sampling temperatures (0.1, 0.2, and 0.3), and the sequence with the lowest global score was selected from each sampling temperature (Design 1, Design 2, and Design 3).

For the designs by ThermoMPNN (https://app.tamarind.bio/tools/thermompnn) (38), using ΔΔG ≤ 0 or −0.5 kcal/mol as the stability threshold for mutations, the mutation with the lowest ΔΔG at each position from saturation mutagenesis was selected as a candidate. All such mutations were compiled into complete protein sequences, designated as ThermoMPNN-design-ΔΔG ≤ 0 kcal/mol (Design 1) and ThermoMPNN-design-ΔΔG ≤ −0.5 kcal/mol (Design 2).

### MD simulation

MD simulation was performed in the absence or presence of Ca^2+^ using the GROMACS software package (version 2020.6) (https://www.gromacs.org/) (76). The protonation state of ionizable residues was set under pH 8.0 based on the pKa values calculated by the H ++ server (http://newbiophysics.cs.vt.edu/H++/index.php) (77). The CHARMM36 force field (78) and the TIP3P water model (79) were used in all simulations. The Ca^2+^-bound and Ca^2+^-unbound structural models were individually solvated in a cubic box with a minimal distance of 1.0 nm from protein to the box edges. Different amounts of Cl^−^ or Na^+^ were added to the system in order to achieve an electro-neutrality system. Initially, energy minimization was accomplished using the steepest descent minimization method until the maximum force was below 1,000 kJ/mol/nm. Afterward, each system was equilibrated by 100 ps MD simulation in the canonical (NVT) ensemble and 250 ps MD simulation in the isothermal–isobaric (NPT) ensemble to allow for the equilibration of the solvent. Finally, all systems underwent three independent 100 ns MD runs without any positional restraints, and the time step of the MD simulations is 2 fs. To keep the experimental conditions consistent, the temperature of the simulation system was constant at 303, 333, or 363 K, and the pressure was stabilized at 1.0 bar using the V-rescale thermostat (80) and Parrinello-Rahman pressure coupling (81, 82). A cut-off of 1.0 nm was used for van der Waals and electrostatic interactions. Long-range electrostatic interactions were estimated using the Particle Mesh Ewald method (83). Bond lengths involving hydrogen atoms were constrained using the LINCS algorithm (84). The time evolution of the RMSF value of the protein backbone was monitored.

## Data Availability

The GenBank accession number of protease C2 is ADD51544.

## SUPPLEMENTAL MATERIAL

Supplemental material aem.01860-25-s0001.pdf Tables S1 to S5; Fig. S1 to S9.

ASM does not own the copyrights to Supplemental Material that may be linked to, or accessed through, an article. The authors have granted ASM a non-exclusive, world-wide license to publish the Supplemental Material files. Please contact the corresponding author directly for reuse.
