# Supplementary material for: Combined computational, rational, and empirical design of boiling-resistant keratinase
Source: Appl Environ Microbiol. 2025 Dec 10;92(1):e01860-25. doi: 10.1128/aem.01860-25 (PMC12838322; doi:10.1128/aem.01860-25)
Supplement: Supplemental material — Tables S1 to S5; Fig. S1 to S9. [file aem.01860-25-s0001.pdf]

Supplementary Material

Combined computational, rational, and empirical design of boiling-resistant keratinase

Yong Yang,[a] Yuewen Luo,[a] Yidi Ding,[a] Yitong Yao,[b] Jie Liu,[a] Zinan Xu,[a] Yu Li,[a] Huai Li,[a] Fei Gan,[a,c] Xiao-Feng Tang,[a,c] # Bing Tang[a,b,c] #

[a]Hubei Key Laboratory of Cell Homeostasis, College of Life Sciences, Wuhan University, Wuhan, China
[b]State Key Laboratory of Virology, College of Life Sciences, Wuhan University, Wuhan, China
[c]Cooperative Innovation Center of Industrial Fermentation (Ministry of Education & Hubei Province), Wuhan, China

Running title: Engineering of hyperthermostable keratinase

#Address correspondence to Bing Tang, tangb@whu.edu.cn or Xiao-Feng Tang, tangxf@whu.edu.cn

Keywords: subtilase, keratinase, thermostability, computational design, rational design, thermophile

TABLE S1 Predicted ion pairs in protease C2 and its variants[a].

		Automated computational design						Structure-based rational design								
	Protease C2	Q20E	N63D	N104D	N105D	Q123D	S163D	N94D	N94E	S163E	S175D	S175E	Y191D	N207D	SM9	CM16
Salt bridge	D56-R101*	√	√	√	√	√	√	√	√	√	√	√	√	√	√	√
	D59-R101*	√	√	√	√	√	√	√	√	√	√	√	√	√	√	√
	D187-K15*	√	√	√	√	×	√	√	√	√	√	√	√	√	×	√
	D187-R269*	√	√	√	√	√	√	√	√	√	√	√	√	√	√	√
	D200-R248*	√	√	√	√	√	√	√	√	√	√	√	√	√	√	√
	E252-R248*	√	√	√	√	√	√	√	√	√	√	√	√	√	√	√
	D256-R269*	√	√	√	√	√	√	√	√	√	√	√	√	√	√	√
	D256-K274*	√	√	√	√	√	√	√	√	√	√	√	√	√	√	√
					D105-R101*											
Long-range ion pair	D23-R26	√	√	√	√	√	√	√	√	√	√	√	√	√	√	√
	D61-R101*	√	√	√	√	√	√	√	√	√	√	√	√	√	√	√
	D66-R101*	√	√	√	√	√	√	√	√	√	√	√	√	√	√	√
	D184-K15*	√	√	√	√	√	√	√	√	√	√	√	√	√	√	√
	D256-K257*	√	√	√	√	×	√	√	√	√	√	√	√	√	√	√
				D5-R9*	D5-R9*	D46-R9		D94-R26*	E94-R26*		D5-R9	E175-R248*	D5-R9*		E94-R26*	E94-R26*
				D46-R9*	D46-R9*	D187-K15*		D94-K50*			D175-R248*		D46-R9*		D175-R248*	D175-R248*
				D104-R101*											D187-K15*	E81-K50

[a]All of the structural models of protease C2 and its variants were predicted by AlphaFold 3. Ion pairs of each protein were analyzed by VMD, and a distance limit of 4 Å and 8 Å was used to define salt bridge and long-range ion pair, respectively. The conserved and disrupted ion pairs in the variants are indicated by "√" and "×", respectively. Newly formed ion pairs are marked in red. The ion pairs involved in ionic networks are indicated by asterisks. The ion pairs of the same ionic network in each protein are shaded with the same color. The single-point variants were constructed by automated computational design or structure based rational design (see Table 1 for details). SM9 (Q20E/S29P/N63D/N94E/Q123D/S130N/S175D/S185P/S219Y) and CM16 (Q20E/S29P/Q41D/N43D/Q48D/N63D/V81E/T86E/N94E/Q123D/S130N/S175D/S185P/N214D/N215D/S219Y) are combination variants.

TABLE S2 Solvent accessibility of amino acid residues of protease C2[a]

Residue	RASA (%)	Loc[b]	Residue	RASA (%)	Loc	Residue	RASA (%)	Loc	Residue	RASA (%)	Loc	Residue	RASA (%)	Loc	Residue	RASA (%)	Loc	Residue	RASA (%)	Loc	Residue	RASA (%)	Loc
Trp1	20.7	O	Val36	0	I	Gly71	0	I	Ser106	83.3	O	Ala141	72.2	O	Gln176	21.3	O	Thr211	0	I	Gln246	63.3	O
Thr2	81.3	O	Asp37	0.5	I	Thr72	0	I	Gly107	37.7	O	Leu142	2.1	I	Ala177	0	I	Tyr212	32.4	O	Ile247	2.9	I
Pro3	0.6	I	Thr38	0.3	I	His73	0	I	Ser108	76.3	O	Gln143	56.6	O	Ile178	0	I	Leu213	26.9	O	Arg248	19.2	I
Asn4	59.8	O	Gly39	0	I	Cys74	0.7	I	Gly109	49.9	O	Ser144	57.9	O	Ala179	0.2	I	Asn214	94.1	O	Ala249	48.5	O
Asp5	0	I	Val40	0	I	Ala75	0	I	Thr110	79.1	O	Ala145	5.3	I	Val180	0	I	Ser215	45.3	O	Ala250	0	I
Leu6	58.2	O	Gln41	13.8	I	Gly76	0	I	Met111	49.1	O	Val146	0	I	Ala181	0	I	Ser216	62.1	O	Ile251	0	I
Thr7	35.6	O	Thr42	25.4	O	Ile77	2.2	I	Ala112	93.4	O	Gln147	54.1	O	Ser182	0	I	Tyr217	17.3	I	Glu252	13.2	I
Ser8	94.7	O	Asn43	77.2	O	Ala78	0	I	Ala113	17.3	I	Gln148	71.3	O	Thr183	0	I	Ala218	31.4	O	Asn253	46	O
Arg9	52.9	O	His44	0.2	I	Ala79	0	I	Val114	7.8	I	Ala149	0	I	Asp184	32.6	O	Ser219	52.5	O	Thr254	10.9	I
Gln10	6.2	I	Pro45	53.9	O	Ala80	0	I	Ala115	10.5	I	Trp150	19	I	Ser185	63.1	O	Leu220	27.6	O	Ala255	0	I
Trp11	21.6	O	Asp46	0	I	Val81	29.2	O	Asn116	59.5	O	Asn151	63.4	O	Asn186	83.9	O	Ser221	36.3	O	Asp256	21.7	O
Gly12	1.2	I	Leu47	0	I	Thr82	0.6	I	Gly117	0	I	Ser152	42.5	O	Asp187	20.1	O	Gly222	2.5	I	Lys257	62.5	O
Pro13	9.3	I	Gln48	56.5	O	Asn83	43.8	O	Ile118	0	I	Gly153	49.7	O	Ser188	66.7	O	Thr223	0.6	I	Ile258	11.5	I
Gln14	42.8	O	Gly49	97.6	O	Asn84	25.9	O	Ala119	41.1	O	Ala154	2.1	I	Leu189	14.2	I	Ser224	17.1	I	Ser259	100	O
Lys15	27.7	O	Lys50	12.4	I	Gly85	49.9	O	Tyr120	19.3	I	Val155	0.2	I	Ser190	0	I	Met225	1.4	I	Gly260	17.8	I
Val16	0	I	Ile51	15.6	I	Thr86	51.1	O	Ala121	0	I	Val156	0	I	Tyr191	88.3	O	Ala226	0	I	Thr261	15.7	I
Gln17	35.7	O	Val52	38.4	O	Gly87	4.1	I	Ala122	5.4	I	Val157	0	I	Phe192	10	I	Thr227	0	I	Gly262	58.7	O
Ala18	0	I	Gln53	69.1	O	Ile88	0.3	I	Gln123	70.6	O	Ala158	0	I	Ser193	0	I	Pro228	0	I	Thr263	77.1	O
Pro19	26.5	O	Gly54	1.4	I	Ala89	1.4	I	Asn124	43.5	O	Ala159	5.2	I	Asn194	4.6	I	His229	0.6	I	Tyr264	42	O
Gln20	49	O	Tyr55	30.1	O	Gly90	0	I	Gly125	58.4	O	Ala160	0	I	Tyr195	31.7	O	Val230	0	I	Phe265	0.2	I
Ala21	0	I	Asp56	2.2	I	Met91	0.1	I	Ala126	0	I	Gly161	14.3	I	Gly196	9.5	I	Ala231	0	I	Gln266	39.2	O
Trp22	0.8	I	Phe57	15.9	I	Ala92	0	I	Asp127	39.3	O	Asn162	45.5	O	Ser197	99.3	O	Gly232	0	I	His267	33.5	O
Asp23	57.8	O	Val58	21.2	O	Pro93	1.4	I	Val128	0	I	Ser163	74.1	O	Trp198	9	I	Leu233	0.1	I	Gly268	0	I
Val24	33.3	O	Asp59	57.2	O	Asn94	54.3	O	Ile129	0	I	Ser164	61.2	O	Val199	0	I	Ala234	0	I	Arg269	5.4	I
Thr25	11.7	I	Asn60	71.2	O	Ala95	0	I	Ser130	1.3	I	Ser165	34.8	O	Asp200	17.5	I	Ala235	0	I	Ile270	0	I
Arg26	40.5	O	Asp61	46.9	O	Ser96	6.7	I	Leu131	0	I	Ser166	67.2	O	Val201	0	I	Leu236	0	I	Asn271	1.1	I
Ser27	0	I	Ser62	77.8	O	Ile97	0	I	Ser132	1.9	I	Thr167	61.1	O	Ala202	0	I	Leu237	0	I	Ala272	0	I
Ser28	45.4	O	Asn63	67.5	O	Met98	2.3	I	Leu133	7.4	I	Pro168	39.5	O	Ala203	0	I	Ala238	11.5	I	Tyr273	47	O
Ser29	96.4	O	Pro64	7.9	I	Pro99	0.7	I	Gly134	46.7	O	Asn169	9.9	I	Pro204	0	I	Ser239	5.2	I	Lys274	53.7	O
Ser30	87.2	O	Gln65	45.9	O	Val100	0	I	Gly135	41.3	O	Tyr170	21.6	O	Gly205	0	I	Gln240	18.7	I	Ala275	0.3	I
Thr31	5.2	I	Asp66	0	I	Arg101	8.7	I	Thr136	81.7	O	Pro171	0.6	I	Ser206	17.6	I	Gly241	81.8	O	Val276	0	I
Val32	13.6	I	Gly67	45.2	O	Val102	0	I	Ser137	79	O	Ala172	0	I	Asn207	82.2	O	Arg242	17.9	I	Asn277	44.8	O
Ile33	0	I	Asn68	23.4	O	Leu103	10.3	I	Gly138	28.7	O	Tyr173	43.2	O	Ile208	5.8	I	Ser243	56.5	O	Tyr278	49.6	O
Ala34	0	I	Gly69	0	I	Asn104	45.1	O	Ser139	31.3	O	Tyr174	7.6	I	Tyr209	17.9	I	Asn244	11.6	I			
Ile35	0.8	I	His70	24.2	O	Asn105	63.1	O	Ser140	100	O	Ser175	92.9	O	Ser210	0	I	Ser245	76	O			

[a]Based on the structure model of protease C2 predicted by AlphaFold 3, the relative solvent accessible surface area (RASA) of the amino acid residue was calculated using GetArea.

[b]The location (Loc) of the residue is considered to be solvent exposed (O) if the RASA value exceeds 20% and to be buried (I) if it is less than 20%.

TABLE S3 Solvent accessibility of ion pairs in Protease C2 and its variants[a].

Enzyme	Interaction[b]	Ion pair	Residue 1	RASA (%) Residue 1	Residue 2	RASA (%) Residue 2	RASA (%) Ion pair	Location[c]
Protease C2	Salt bridge	D56-R101	D56	2.2	R101	8.7	5.45	I
		D59-R101	D59	57.2	R101	8.7	32.95	O
		D187-K15	D187	20.1	K15	27.7	23.9	O
		D187-R269	D187	20.1	R269	5.4	12.75	I
		D200-R248	D200	17.5	R248	19.2	18.35	I
		E252-R248	E252	13.2	R248	19.2	16.2	I
		D256-R269	D256	21.7	R269	5.4	13.55	I
		D256-K274	D256	21.7	K274	53.7	37.7	O
	Long-range ion pair	D23-R26	D23	57.8	R26	40.5	49.15	O
		D61-R101	D61	46.9	R101	8.7	27.8	O
		D66-R101	D66	0	R101	8.7	4.35	I
		D184-K15	D184	32.6	K15	27.7	30.15	O
		D256-K257	D256	21.7	K257	62.5	42.1	O
N94D	Long-range ion pair	D94-R26	D94	52.6	R26	40.4	46.5	O
		D94-K50	D94	52.6	K50	12.9	32.75	O
N94E	Long-range ion pair	E94-R26	E94	55.6	R26	37.4	46.5	O
S175D	Long-range ion pair	D5-R9	D5	1	R9	52.5	26.75	O
		D175-R248	D175	92.9	R248	19.2	56.05	O
S175E	Long-range ion pair	E175-R248	E175	85.5	R248	19.4	52.45	O
Y191D	Long-range ion pair	D5-R9	D5	0.4	R9	49.5	24.95	O
		D46-R9	D46	0	R9	49.5	24.75	O

[a] Based on the structure models of protease C2 and its variants predicted by AlphaFold 3, the relative solvent accessible surface area (RASA) of the amino acid residue was calculated using GetArea. The RASA of an ion pair is the average of the RASA values of the two residues forming the ion pair.

[b]For the variants, only the newly formed ion pairs are shown

[c]The residue is considered to be solvent exposed (O) if the RASA value exceeds 20% and to be buried (I) if it is less than 20%.

TABLE S4 Predicted Type I β-turns in protease C2[a]

Number	Turn	Sequence[b]	Pro substitution	Note
1	Ser28-Thr31	SSST	S29P	Automated computational design
2	Gln41-His44	QTNH	T42P	
3	His44-Leu47	HPDL		
4	Phe57-Asn60	FVDN		Ca^{2+}-binding site (Ca2)
5	Ala92-Ala95	APNA		
6	Asn104-Gly107	NNSG	N105P	
7	Pro171-Tyr174	PAYY	A172P	
8	Tyr174-Ala177	YSQA	S175P	Automated computational design
9	Asp184-Asp187	DSND	S185P	
10	Ser190-Ser193	SYFS	Y191P	
11	Gly196-Val199	GSWV	S197P	Automated computational design
12	Phe265-Gly268	FQHG	Q266P	

[a]The structure model of protease C2 was predicted by AlphaFold 3 and then analyzed using PDBsum to identify the type I β-turns.

[b]The residues at the *i* + 1 positions of the β-turns are marked in red.

TABLE S5 Primers used in this study [a]

Primer	Nucleotide sequence (5' to 3')	Protein or mutation
pro-C2-F	CTTTAAGAAGGAGATATACATATGGCCAGCCCTGCCTC	Protease C2 proform
pro-C2-R	CGACGGAGCTCGAATTCTTAGTGGTGGTGGTGGTGGTGATAATTGACG	
L6F-F	CAATGACTTTACCAGTCGCCAATGG	L6F
L6F-R	GACTGGTAAAGTCATTGGGTGTCCA	
K15A-F	CCGCAAGCCGTCCAAGCTCCCCAA	K15A
K15A-R	CTTGGACGGCTTGCGGTCCCCATTG	
Q20E-F	TCCAAGCTCCCGAAGCTTGGGATG	Q20E
Q20E-R	CATCCCAAGCTTCGGGAGCTTGGA	
R26A-F	TGTCACCGCCAGCAGCAGCAGCACG	R26A
R26A-R	CTGCTGCTGGCGGTGACATCCCAAGC	
S27G-F	CCCGCGGCAGCAGCAGCACGGTG	S27G
S27G-R	CTGCTGCCGCGGGTGACATCCCA	
S29P-F	AGCAGCCCAAGCACGGTGATTGCCA	S29P
S29P-R	CGTGCTTGGGCTGCTGCGGGTGAC	
Q41D/N43D-F	ACACCGGAGTCGATACCGATCATCCGGAT	Q41D/N43D
Q41D/N43D-R	ATCCGGATGATCGGTATCGACTCCGGTGT	
T42P-F	CCGGAGTCCAACCCAATCATCCGG	T42P
T42P-R	CCGGATGATTGGGTTGGACTCCGG	
Q48D-F	CATCCGGATTTGGATGGAAAAATCGT	Q48D
Q48D-R	ACGATTTTTCCATCCAAATCCGGATG	
K50R-F	CAGGGACGTATCGTCCAAGGGTAT	K50R
K50R-R	GACGATACGTCCCTGCAAATCCG	
D56N-F	GGGTATAACTTTGTCGATAACGACA	D56N
D56N-R	GACAAAGTTATACCCTTGGACGAT	
D59K-F	TTGTCAAAAACGACAGCAATCCC	D59K
D59K-R	TGTCGTTTTTGACAAAGTCATACCC	
D59R-F	TTGTCCATAACGACAGCAATCCC	D59R
D59R-R	TGTCGTTATGGACAAAGTCATACCC	
D59N-F	TTGTCAATAACGACAGCAATCCC	D59N
D59N-R	TGTCGTTATTGACAAAGTCATACCC	
D61N-F	GATAACAACAGCAATCCCCAAGAC	D61N
D61N-R	ATTGCTGTTGTTATCGACAAAGT	
N63D-F	ATAACGACAGCGATCCCCAAGACG	N63D
N63D-R	CGTCTTGGGGATCGCTGTCGTTAT	
C74V-F	TGGAACCCACGTCGCAGGAATTGC	C74V
C74V-R	CCTGCGACGTGGGTTCCATGTCC	
V81D-F	GCTGCCGATACCAATAACGGAACG	V81D
V81D-R	TTGGTATCGGCAGCGGCAATTCCTGCGC	
V81N-F	GCTGCCAATACCAATAACGGAACG	V81N
V81N-R	TTGGTATTGGCAGCGGCAATTCCTGCGC	
V81E-F	TGCCGCTGCCGAGACCAATAACG	V81E
V81E-R	CGTTATTGGTCTCGGCAGCGGCA	

V81Q-F	GCTGCCCAGACCAATAACGGAACG	V81Q
V81Q-R	TTGGTCTGGGCAGCGGCAATTCCTGCGC	
G85D-F	CAATAACGACACGGGAATTGCAGGA	G85D
G85D-R	ATTCCCGTGTCGTTATTGGTCACGGC	
G85N-F	CAATAACAATACGGGAATTGCAGGA	G85N
G85N-R	ATTCCCGTATTGTTATTGGTCACGGC	
G85E-F	CAATAACGAAACGGGAATTGCAGGAATGGCTCCCA	G85E
G85E-R	ATTCCCGTTTCGTTATTGGTCACGGCAGCGG	
G85Q-F	CAATAACCAAACGGGAATTGCAGGA	G85Q
G85Q-R	ATTCCCGTTTGGTTATTGGTCACGGC	
T86D-F	ACGGAGATGGAATTGCAGGAATG	T86D
T86D-R	ATTCCATCTCCGTTATTGGTCAC	
T86N-F	ACGGAAATGGAATTGCAGGAATG	T86N
T86N-R	ATTCCATTTCCGTTATTGGTCAC	
T86E-F	ACCAATAACGGAGAGGGAATTGCAGG	T86E
T86E-R	CCTGCAATTCCCTCTCCGTTATTGGT	
T86Q-F	ACGGACAGGGAATTGCAGGAATG	T86Q
T86Q-R	ATTCCCTGTCCGTTATTGGTCAC	
N94E-F	GGCTCCCGAAGCCTCCATCATGCCG	N94E
N94E-R	GGAGGCTTCGGGAGCCATTCCTGC	
N94D-F	GCTCCCGACGCCTCCATCATGCCGG	N94D
N94D-R	GGAGGCGTCGGGAGCCATTCCTGC	
N104D-F	GTTCGCGTGCTGGATAACAGCGGAAG	N104D
N104D-R	CTTCCGCTGTTATCCAGCACGCGAAC	
N105D-F	GCGTGCTGAATGACAGCGGAAGCGGA	N105D
N105D-R	CCGCTTCCGCTGTCATTCAGCACGC	
N105P-F	GCGTGCTGAATCCCAGCGGAAGCGGA	N105P
N105P-R	CCGCTTCCGCTGGGATTCAGCACGC	
S106N-F	GTGCTGAATAACAACGGAAGCGGA	S106N
S106N-R	TCCGCTTCCGTTGTTATTCAGCAC	
M111L-F	GAAGCGGAACCCTGGCAGCCGTTG	M111L
M111L-R	GCAACGGCTGCCAGGGTTCCGCTT	
A112S-F	GCGGAACCATGTCAGCCGTTGCC	A112S
A112S-R	GGCAACGGCTGACATGGTTCCGC	
A113W-F	ATGGCATGGGTTGCCAACGGAATC	A113W
A113W-R	GGCAACCCATGCCATGGTTCCGCT	
V114I-F	ACCATGGCAGCCATTGCCAACGGAAT	V114I
V114I-R	TTCCGTTGGCAATGGCTGCCATGG	
A119I-F	GAATCATTTATGCCGCCCAAAAC	A119I
A119I-R	CGGCATAAATGATTCCGTTGGCAACGGCTG	
Q123D-F	GCTTATGCCGCCGATAACGGGGCAGA	Q123D
Q123D-R	TCTGCCCCGTTATCGGCGGCATAAGC	
S130N-F	GTCATCAACTTGAGCTTGGGTGGC	S130N
S130N-R	GCTCAAGTTGATGACATCTGCCC	

G135S-F	CTTGAGCTTGGGTTCCACTTCCGGAA	G135S
G135S-R	TTCCGGAAGTGGAACCCAAGCTCAAG	
S137T-F	TGGGTGGCACTACCGGAAGCTCGG	S137T
S137T-R	CCGAGCTTCCGGTAGTGCCACCCAA	
A141T-F	AGCTCGACCTTGCAAAGCGCCGTT	A141T
A141T-R	TTGCAAGGTCGAGCTTCCGGAAGT	
Q148Y-F	AGCGCCGTTCAATATGCTTGGAACAG	Q148Y
Q148Y-R	CTGTTCCAAGCATATTGAACGGCGCT	
S152K-F	GGAACAAAGGAGCTGTCGTCGTT	S152K
S152K-R	CAGCTCCTTTGTTCCAAGCTTGTTG	
S163D-F	CTGCAGGAAACGACAGCAGCTCCA	S163D
S163D-R	TGGAGCTGCTGTCGTTTCCTGCAG	
S163E-F	CCGCTGCAGGAAACGAGAGCAGCTCCAC	S163E
S163E-R	GTGGAGCTGCTCTCGTTTCCTGCAGCGG	
S164G-F	ACAGCGGCAGCTCCACTCCCAAC	S164G
S164G-R	TGGAGCTGCCGCTGTTTCCTGCAGC	
S164N-F	AGGAAACAGCAACAGCTCCACTC	S164N
S164N-R	GAGTGGAGCTGTTGCTGTTTCCT	
A172P-F	CCAACTATCCGCCTTATTACTCT	A172P
A172P-R	AGAGTAATAAGGCGGATAGTTGG	
S175D-F	CCGGCTTATTACGATCAAGCCATTG	S175D
S175D-R	CAATGGCTTGATCGTAATAAGCCG	
S175E-F	CCGGCTTATTACGAGCAAGCCATTGCT	S175E
S175E-R	AGCAATGGCTTGCTCGTAATAAGCCGG	
S175P-F	CCGGCTTATTACCCTCAAGCCATT	S175P
S175P-R	AATGGCTTGAGGGTAATAAGCCG	
Q176N-F	ACTCTAATGCCATTGCTGTCGCT	Q176N
Q176N-R	CAATGGCATTAGAGTAATAAGCCGG	
A177V-F	TCTCAAGTCATTGCTGTCGCTTCC	A177V
A177V-R	AGCAATGACTTGAGAGTAATAAGC	
S182A-F	GTCGCTGCCACCGATTCCAATGAC	S182A
S182A-R	ATCGGTGGCAGCGACAGCAATG	
S185P-F	GCTTCCACCGATCCCAATGACAGC	S185P
S185P-R	CTGTCATTGGGATCGGTGGAAGC	
S190A-F	AGCCTGGCTTACTTCTCCAACTAC	S190A
S190A-R	GAAGTAAGCCAGGCTGTCATTGGA	
Y191D-F	CTGTCTGACTTCTCCAACTACGGA	Y191D
Y191D-R	GGAGAAGTCAGACAGGCTGTCATTGG	
Y191P-F	GACAGCCTGTCTCCCTTCTCCAACTA	Y191P
Y191P-R	TAGTTGGAGAAGGGAGACAGGCTGT	
Y191S-F	CTGTCTTCCTTCTCCAACTACGGA	Y191S
Y191S-R	GGAGAAGGAAGACAGGCTGTCATT	
S197P-F	CTACGGACCCTGGGTGGATGTAGCC	S197P
S197P-R	ACCCAGGGTCCGTAGTTGGAGAA	

S206V-F	CCGGGTGTCAACATCTACTCCACC	S206V
S206V-R	GATGTTGACACCCGGAGCGGCTAC	
N207D-F	GCTCCGGGTTCCGACATCTACTCC	N207D
N207D-R	GGAGTAGATGTCGGAACCCGGAGC	
Y212W-F	CTACTCCACCTGGCTCAACAGCA	Y212W
Y212W-R	TGCTGTTGAGCCAGGTGGAGTA	
N214D/S215D-F	CCACCTACCTCGACGACAGTTATGCA	N214D/S215D
N214D/S215D-R	TGCATAACTGTCGTCGAGGTAGGTGG	
S219Y-F	GTTATGCATATTTGAGCGGAACCTCCATG	S219Y
S219Y-R	CGCTCAAATATGCATAACTGCTGTTG	
L220M-F	TTATGCATCCATGAGCGGAAC	L220M
L220M-R	GTTCCGCTCATGGATGCATAA	
Q266P-F	ATCAGCGGCACCGGAACTTACTTCCCACACGGAAGAATC	Q266P
Q266P-R	GATTCTTCCGTGTGGGAAGTAAGTT	
Ca5M-1-F	ATACAGCCGATGATCTCGGCCCCACCGGAACT	Ca5M
Ca5M-1-R	AGTTCCGGTGGGGCCGAGATCATCGGCTGTAT	
Ca5M-2-F	CCCCACCGGATGGGACGCCGATTACGGAAGAATC	
Ca5M-2-R	TGATTCTTCCGTAATCGGCGTCCCATCCGGTGGGG	

[a] Underlined sequences indicate restriction enzyme sites. The shaded section indicates the His6 tag-coding DNA sequence. The mutated nucleotides are boxed.

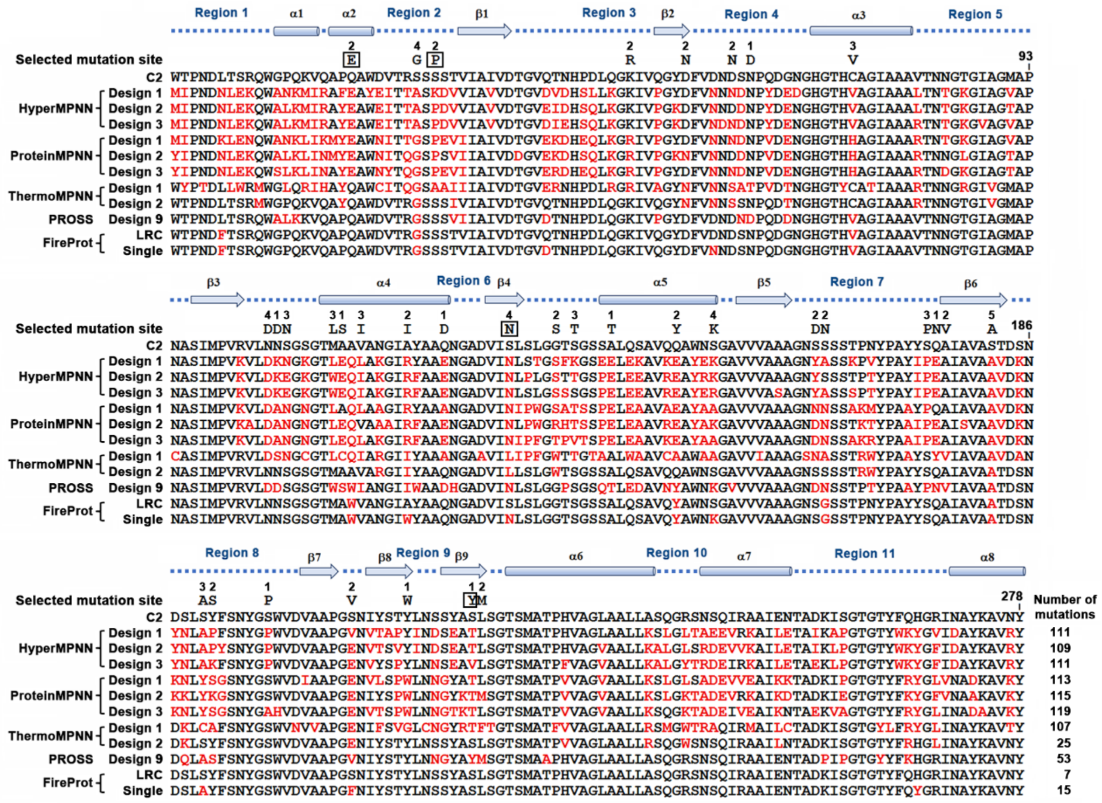

FIG S1 Amino acid sequences of the stabilizing variants of protease C2 designed by HyperMPNN, ProteinMPNN, ThermoMPNN, PROSS, and FireProt. The mutation sites in the designed variants are marked in red. The mutations selected for construction of single-point variants in this study are indicated, and the experimentally confirmed stabilizing mutations are boxed. The numbers above the selected mutation sites represent the times predicted by the five computational tools. The α-helices (α1 to α8), β-strands (β1 to β9), and the regions (regions 1 to 11) between or outside of the secondary structures are shown above the alignment. The total number of mutations in each design is indicated at the bottom right corner.

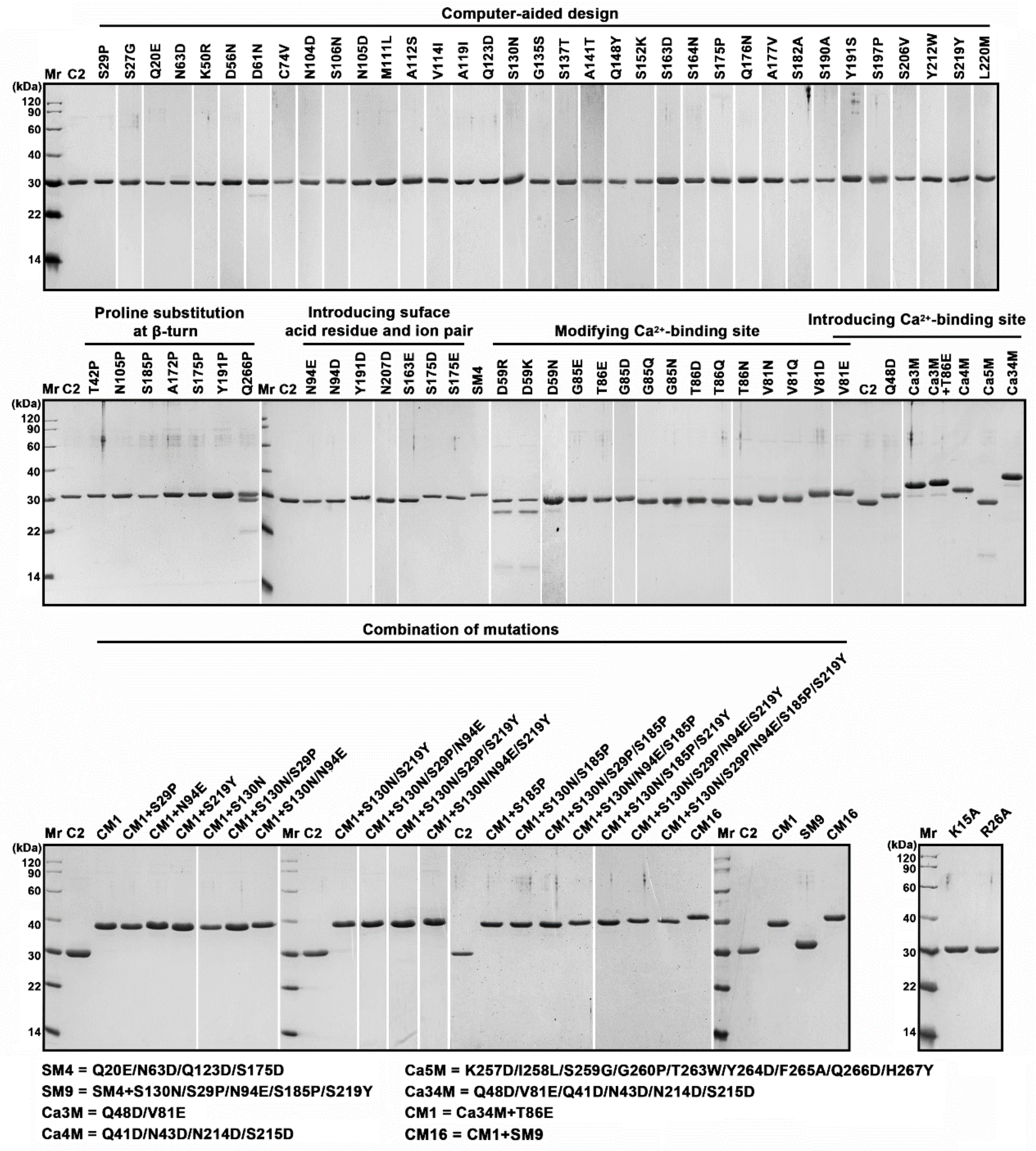

FIG S2 SDS-PAGE analysis of purified enzyme samples. Note that although protease C2 and its variants have similar theoretical molecular weight, some variants showed higher apparent molecular weight than that of protease C2 by SDS-PAGE analysis. This is because these variants possess a higher content of acidic residues than protease C2, and the overestimation of molecular weight by SDS-PAGE is a common feature of acidic proteins (Madern D, Ebel C, Zaccai G. 2000. Halophilic adaptation of enzymes. Extremophiles 4:91–98).

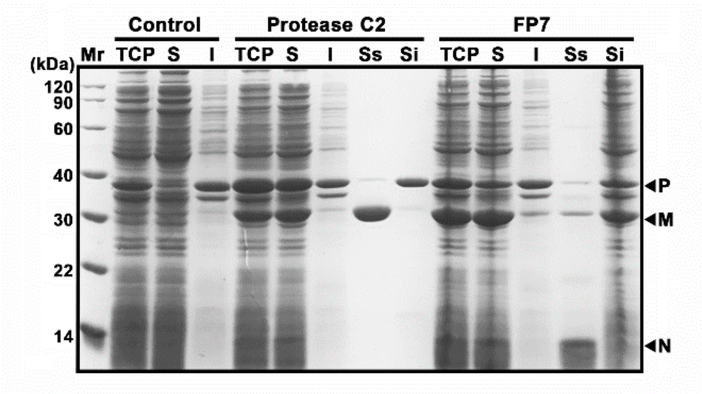

FIG S3 Production and maturation of recombinant protease C2 and its variant FP7 (L6F/S27G/C74V/A113W/Q148Y/S164G/S182A). The total cellular protein (TCP), the soluble (S) and insoluble (I) fractions of *E. coli* cells harboring a blank vector (control) or producing recombinant proform of the enzyme, as well as the soluble (Ss) and insoluble (Si) fractions of the heat-treated (85°C, 15 min) soluble fraction (S) of *E. coli* cells was subjected to SDS-PAGE analyses. The bands corresponding to the proform (P), the mature form (M), and the processed N-terminal propeptide (N) are indicated.

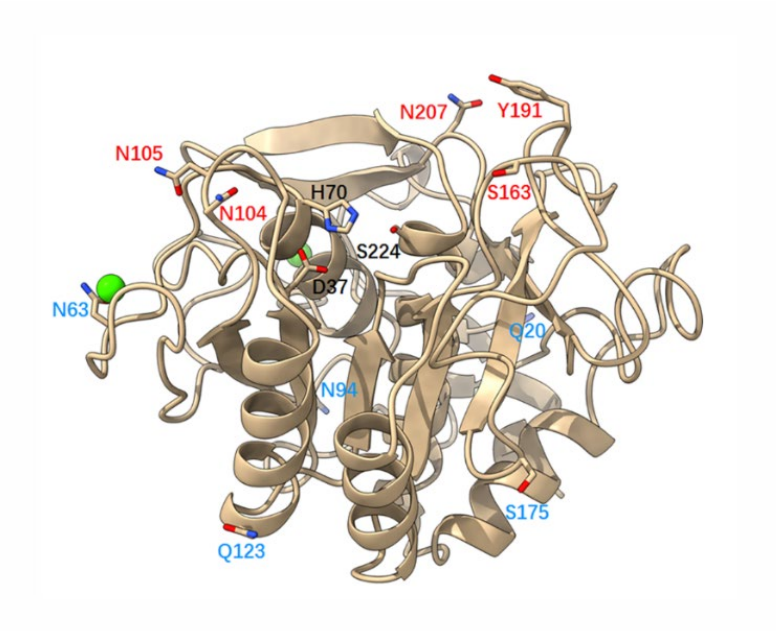

FIG S4 Structural model of protease C2 predicted by AlphaFold 3. The catalytic residues (D37, H70, and S224) and the residues for Asp/Glu substitution, selected either by automated computational design (Q20, N63, N104, N105, Q123 and S163D; Table 1) or by manually checking whether the substitution has the potential to form (N94, S175, and Y191) an ion pair or not (S163 and N207), are indicated. The residues far away from the active site are marked in blue and those close to the active site are marked in red.

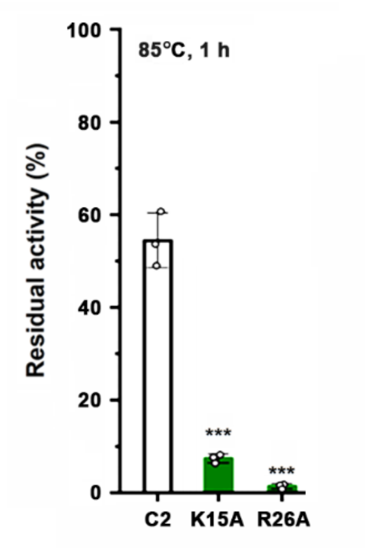

FIG S5 Thermal resistance of the enzymes. The enzymes (1 μg/mL) were incubated at 85°C for 1 h in 50 mM Tris-HCl (pH 8) containing 10 mM $CaCl_2$ and then subjected to azocaseinolytic activity assay at 60°C. The residual activity is expressed as a percentage of the original activity. The values are expressed as means ± SDs from three independent experiments (***, P <0.001; calculated by Student's *t* test).

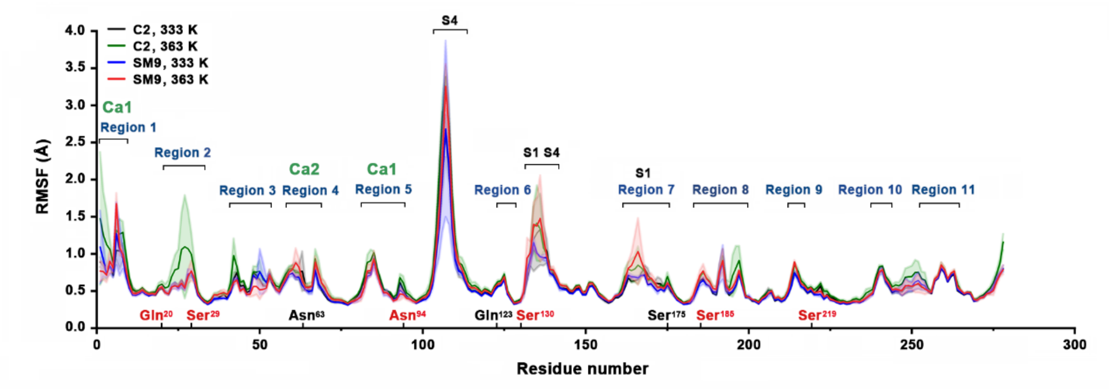

FIG S6 MD simulations of the Ca^{2+}-bound forms of protease C2 and SM9 at different temperatures (333 K and 363 K). The RMSF values are expressed as means ± SDs (shaded area) of three independent 100-ns MD trajectories for each enzyme. The regions 1 to 11, the substrate binding sites (S1 and S4), and the Ca^{2+}-binding sites (Ca1 and Ca2) of the enzyme are indicated. The positions of the residues that were mutated in SM9 are shown. The mutation sites of the single-point variants with improved thermostability are marked in red.

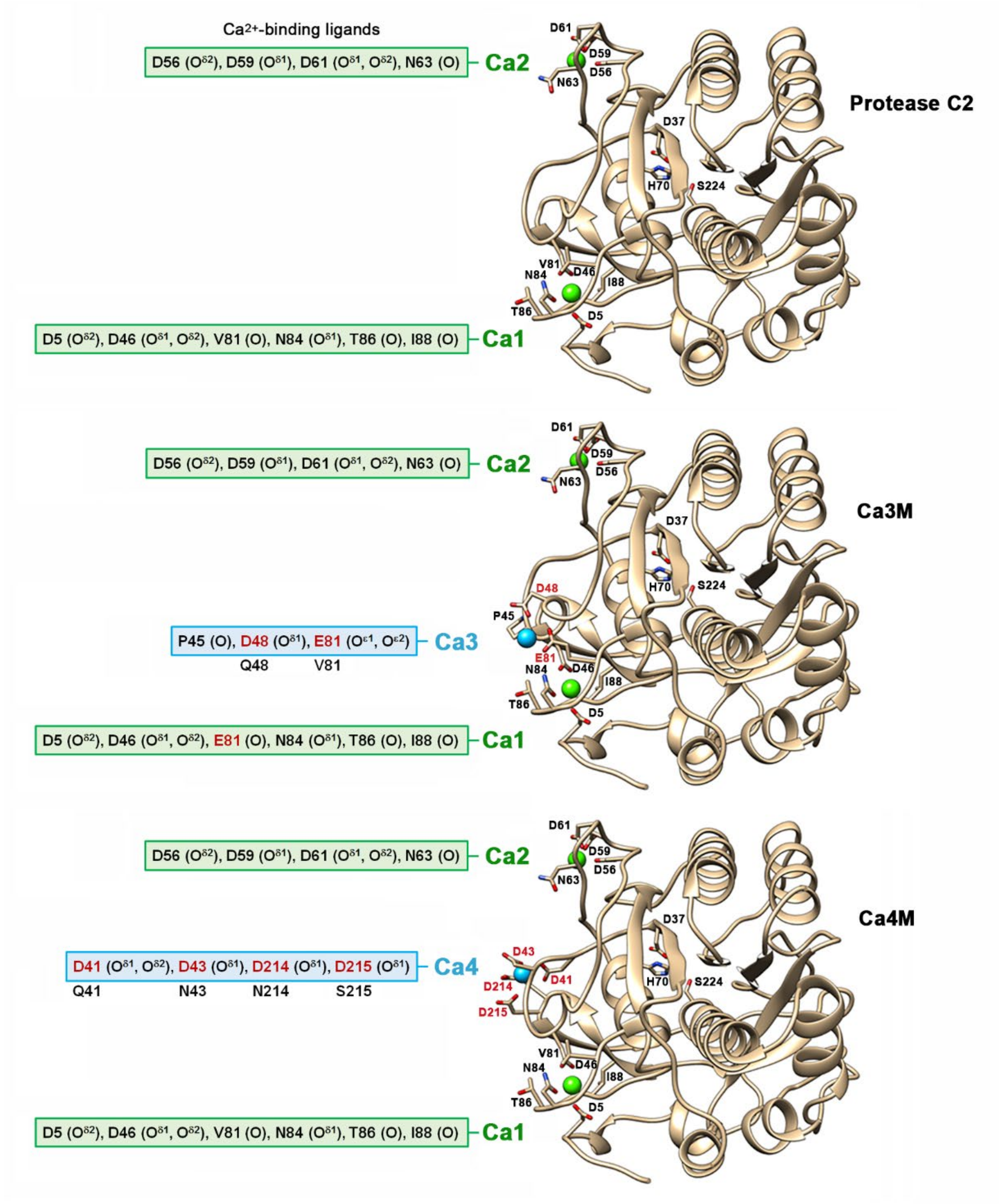

FIG S7 The Ca^{2+}-binding ligands in the structural models of protease C2 and its variants Ca3M and Ca4M. All of the structural models were predicted by AlphaFold 3. The Ca^{2+}-binding ligands are shown, and the mutated residues were marked in red. The catalytic residues (D37, H70, and S224) are indicated.

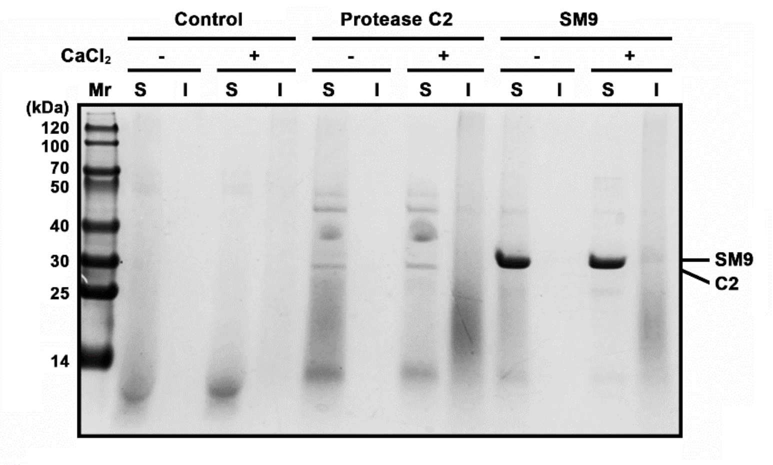

FIG S8 Effect of $CaCl_2$ on the solubility of the degradation products of feather. In the absence (control) or presence of protease C2 or SM9 (2 μg/ml), feather meal (3 mg/ml) was incubated at 70°C for 4 h in 1.5 ml of 50 mM Tris-HCl (pH 8) containing 0.5% β-Me. After centrifuging the reaction mixture at 13,400 × *g* for 10 min, the supernatant was supplemented with (+) or without (-) 10 mM $CaCl_2$ and then incubated at 70°C for 30 min, followed by centrifugation at 13,400 × *g* for 10 min to recover the soluble (S) and insoluble (I) fractions for Tricine-SDS-PAGE analysis. Note that the presence of $CaCl_2$ led to the precipitation of degradation products of feather (lanes I). The positions of protease C2 and SM9 on the gel are indicated. Because SM9 is more thermostable than protease C2, after the degradation reaction a higher amount of SM9 remained compared with protease C2.

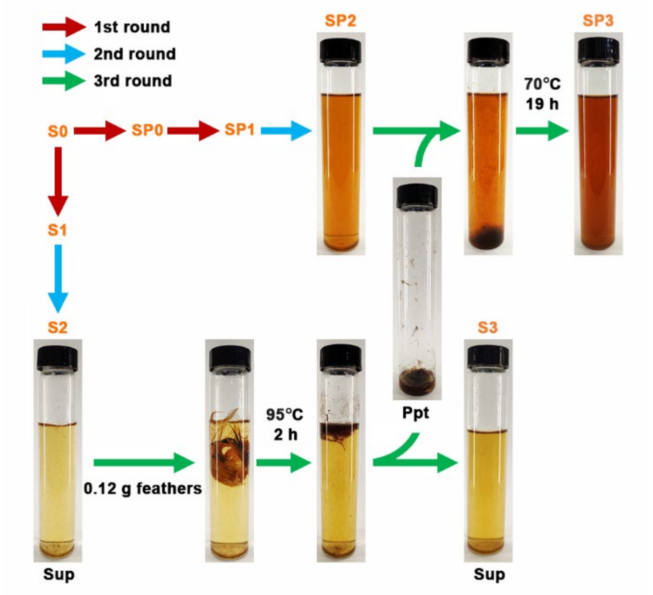

FIG S9 The third round of degradation of native chicken feather by SM9 via enzyme recycling. The reaction was carried out at the indicated conditions, and the supernatant (Sup) and pellet (Ppt) of the reaction mixture were separated by centrifugation at 13,400 × *g* for 10 min (A). The samples of the first-round (S0, SP0, S1, and SP1) and second-round (S2 and SP2) reactions are the same as those shown in Fig. 8.
